# Non-nodulating *Paenirhizobium* fix nitrogen in association with cereal roots

**DOI:** 10.1093/ismeco/ycag156

**Published:** 2026-06-24

**Authors:** Matthew J Campbell, Beatriz Jorrin, Zohar Katz, Andrzej Tkacz, Raphael Ledermann, Philip S Poole

**Affiliations:** Department of Biology, University of Oxford, South Parks Road, OX1 3EL, Oxford, United Kingdom; Department of Biology, University of Oxford, South Parks Road, OX1 3EL, Oxford, United Kingdom; Department of Biology, University of Oxford, South Parks Road, OX1 3EL, Oxford, United Kingdom; Department of Biology, University of Oxford, South Parks Road, OX1 3EL, Oxford, United Kingdom; Department of Biology, University of Oxford, South Parks Road, OX1 3EL, Oxford, United Kingdom; Department of Biology, University of Oxford, South Parks Road, OX1 3EL, Oxford, United Kingdom

**Keywords:** biological nitrogen fixation (BNF), free-living diazotrophs, *Paenirhizobium, nif-fix* genes, acetylene reduction assays (ARAs)

## Abstract

Cereal root microbiomes harbour diverse diazotrophic bacteria, yet the taxa capable of sustained nitrogen fixation in association with cereal roots remain poorly characterized. Here, two high-performing nitrogen-fixing strains (B6 and J2) were isolated from barley roots and identified as belonging to the family Rhizobiaceae in the genus *Paenirhizobium*. Both strains possess plasmid-encoded canonical rhizobial *nif* and *fix* genes for nitrogen fixation but lack nodulation genes. Their genomes have a 5.7 Mb chromosome and four *repABC* plasmids. Unlike most nodulating rhizobia, strains B6 and J2 fixed nitrogen in laboratory culture on a range of carbon sources, achieving maximal activity on organic acids at low ammonium (<0.5 mM) and oxygen concentrations (1–3%). Both strains colonized the total root systems of barley plants, with population densities of 10^6^ CFU g^−1^ fresh root weight. Strains fixed high levels of nitrogen on barley plants, similar to or greater than other known free-living diazotrophs. These findings expand the ecological context of rhizobial nitrogen fixation and identify cereal-associated *Paenirhizobium* as a previously unrecognized component of the diazotrophic cereal root microbiome.

## Introduction

Globally, terrestrial biological nitrogen fixation (BNF), performed by symbiotic, free-living and associative bacteria, contributes more N to soils than any other process, supplying between 52 and 140 Tg N yr^−1^ [1]. While symbiotic partnerships like those between legumes and rhizobia dominate global BNF inputs, contributing roughly two-thirds of this total N input, free-living and associative N_2_-fixing bacteria remain vital contributors, accounting for 33% of terrestrial fixation [2, 3]. Importantly, many of these bacteria colonize the rhizosphere and rhizoplane of non-leguminous plants, including cereal crops, and do not require specialized symbiotic structures to fix N_2_, such as nodules found in the rhizobia-legume symbioses [4, 5].

Isolation of diazotrophs from soil is highly dependent on the selective N-free culturing medium (solid or semi-solid), as auxotrophic strains will require additional nutrients to facilitate growth [6, 7]. Specialized media like universal minimal salts (UMS) media [8], contain a range of micronutrients and B-vitamins, favouring the growth of rhizobia and other plant-associated specialists which are often auxotrophs [9]. Notably, the rhizocompartments of wheat and faba beans tend to harbour reduced microbial diversity relative to the surrounding bulk soil, with bacteria persisting here better adapted to intimate plant–microbe interactions [10, 11]. Targeted isolation from these regions, enriched with diazotrophs naturally pre-adapted for growth and persistence on cereal crop roots, would provide an important reservoir for potential plant growth promoting (PGP) strains.

Here, we describe the isolation of two diazotrophic *Paenirhizbium* strains from barley roots using rhizobial media UMS and identified them using whole-genome sequencing. We evaluated their N_2_-fixing potential both under free-living conditions and in association with barley roots. By characterizing their growth on different carbon sources, sensitivity to environmental regulators, and colonization ability, we position these strains as model diazotrophs isolated specifically from cereal crop roots.

## Materials and methods

### Bacterial strains and conditions

Bacteria used in this study are in Supplementary Table 1. Rhizobial strains were streaked onto tryptone-yeast (TY) 1.75% agar [12] on plates or 10 ml slopes and incubated at 28°C for 2–3 days. All *Escherichia coli* strains were grown on LB 1.75% agar [13] at 37°C overnight. UMS [8, 14] was supplemented with vitamins (1 μg ml^−1^ thiamine hydrochloride, 0.2 μg ml^−1^ D-pantothenic acid, 0.1 μg ml^−1^biotin) and 10 mM ammonium chloride (NH_4_Cl). Carbon sources were added at the following concentrations: 10 mM glucose (UMS^Gluc^), 20 mM succinate (UMS^Succ^), 30 mM sodium pyruvate, 10 mM sucrose, 20 mM L-malic acid, or 10 mM methanol (UMS^MeOH^). Agar (1.75% w/v) was added to universal minimal agar (UMA). For plasmid-containing strains, antibiotics were added at the following concentrations: nitrofurantoin (10 μg ml^−1^), gentamicin (20 μg ml^−1^), kanamycin (50 μg ml^−1^), and neomycin (80 μg ml^−1^).

### Initial isolation of diazotrophs from barley roots

Unsterilized barley seeds (*Hordeum vulgare* cv. Golden Promise) were sown into pots containing a 1:1 mixture of unsterilized organic-rich soil from Wytham forest (Oxford, UK) [15] and F2 commercial compost (Levington). Plants were harvested after 14 days growth before soil was removed from roots, which were washed with sterile water, dried and cut into 1 g sections. These were ground using a sterile pestle and mortar and resuspended in phosphate-buffer saline (PBS; Merck, Germany). Suspensions were centrifuged at 1000 rpm for 5 min to remove plant debris, before spinning at 5000 rpm to pellet bacteria and resuspending in 2 ml UMS for storage in 20% (v/v) glycerol at −80°C.

### Cultivation of root-associated diazotrophs

Diazotrophs were isolated as pellicles in semi-solid N-free UMS medium (0.175% w/v agarose) supplemented with succinate, glucose, or methanol. Universal tubes containing 20 ml semi-solid UMS were stab-inoculated with root-derived samples and incubated at 28°C for 7–14 days until pellicle formation. Pellicles from identical conditions were combined into a single culture and inoculated into 25 ml liquid UMS in 250 ml flasks and incubated under microaerobic conditions (1% O₂, ~99% N₂ headspace) in an oxygen cabinet at 28°C and shaking at 130 rpm for ~7 days. To isolate individual strains, cultures were serially diluted and spread onto TY agar plates. Colonies from the TY plates were inoculated into N-free liquid UMS cultures and incubated in O₂ microaerobic conditions (3% O_2_) at 28°C and 130 rpm until growth was observed (~7 days).

### Colony PCR

DNA was extracted from bacteria grown in N-free media and used for colony PCR using primers IKG3/DVV [16] to amplify 16S rRNA and nitrogenase subunit *nifH* (Supplementary Table 2).

### Genome sequencing

B6 and J2 were sequenced by Microbes NG (Birmingham, UK) on the MiSeq platform (v3 chemistry, paired end 2× 250 bp) and Oxford Nanopore sequencing (utilized R10.4.1 chemistry for long reads). Raw reads were assembled with HybridSpades (v3.15.3) [17], circularized with Unicycler (v0.4.8) [18] and annotated with RASTtk (v1.073) [19].

### Whole genome synteny

Genomes were aligned using Nucmer (v3.1) [20]. The resulting .*coords* file was processed for compatibility with Circos (v0.69–8) [21] for visualizing genome synteny. Genomic regions with <96% nucleotide identity were classified as low-identity and visualized with black lines.

### Average amino acid identity and average nucleotide identity

Proteomes of genomes (Supplementary Table 3) were downloaded from NCBI and JGI in March 2025. Pairwise average amino acid identity (AAI) was calculated using EzAAi [22] with minimum identity and coverage thresholds of 0.3 and 0.7, respectively (Supplementary Table 4) [23]. Pairwise average nucleotide identity (ANI) was calculated using FastANI [24]. Pairwise AAI and ANI outputs were converted into a matrix using *list2matrix.py* and transformed into a lower triangular dissimilarity matrix using *sim2distlow.py* (Supplementary Methods), resulting in a lower triangular matrix containing the dissimilarity values for each strain pair. The matrix was imported into MEGA X [25] to construct a UPMGA tree (Supplementary Fig. 1).

### NifH taxonomy

NifH protein sequences from related strains were aligned using Clustal Omega (v 1.2.4) (Supplementary Material) [26]. A maximum likelihood tree was calculated with IQ-TREE (v2.3.6) [27], with branches collapsed to 70% using stream editor option *nw_ed* from Newick Utilities (1.6) [28]. Trees were visualized using Figtree (v1.4.4) and Inkscape (v1.4).

### Nif-fix synteny

Genomic regions containing *nif* and *fix* genes were extracted in GenBank format and analysed with CAGECAT [29]. Gene cluster comparisons were generated using the *clinker* option with an identity threshold of 0.5.

### Biolog™ phenotyping plates

Rhizobial strains were grown in UMS^Succ^ in baffled flasks three days prior to phenotyping. Cultures were washed three times in UMS, transferred to fresh baffled flasks containing UMS supplemented with NH₄Cl and vitamins, and carbon-starved for 6 h. Cells were adjusted to OD_600 nm_ = 0.1 and mixed with 0.1% (w/v) 2, 3, 5-triphenyltetrazolium chloride (TTC) before inoculating into PM1 Biolog Phenotype Microarray™ plates (Biolog, USA). Plates were incubated in an Omega FluoStar plate reader (BMG Labtech, Germany) shaking at 500 rpm, with absorbance measured at 505 and 595 nm every 15 minutes for 50 h. Growth curves were visualized using MARS software (BMG Labtech, Germany) and mean generation times (MGTs) were calculated as the time taken for the population to double during exponential growth phase.

### Diazotrophic growth curves

Single colonies were streaked onto TYA slopes and incubated for two days. Slopes were washed three times and inoculated at OD_600nm_ = 0.02 into baffled flasks containing 5 ml N-free UMS^Succ^. Cultures were adjusted to 1% O_2_ microaerobic environment and left shaking to grow for 10 days, with changes in optical density measured every 12 h.

### Bacterial conjugations

Chromosome-integrated mini-Tn7-GFP derivatives of strains B6 and J2 were generated via triparental mating. *E. coli* strains carrying pRK2013 [30] and the pTNS3 transposase plasmid to assist conjugation efficiency and transfer of pTn7-SCOUT11.G. Transconjugants were selected on TY agar plates containing nitrofurantoin and gentamicin with successful integration confirmed by PCR.

### Free-living and *in-planta* acetylene reduction assays

For free-living assays, strains were grown on TYA slopes, washed three times with PBS, and inoculated at OD_600 nm_ = 0.3 into sterile glass universals with N-free UMS. Cultures were equilibrated under microaerobic conditions (1% O_2_) before 10% of the headspace was replaced with 10% acetylene. After 24 hr incubation at 28°C with shaking (130 rpm), 1 ml gas samples were analysed on a PerkinElmer Clarus 480 gas chromatograph with a HayeSep® N (80–100 MESH) 584 column at 150°C and a carrier gas flow rate of 20 ml/minute. Biological replicates were normalized by protein content using bovine serum albumin [31].

*In-planta* acetylene reduction assays (ARAs) and barley seed pre-germination followed methods outlined in [32]. N_2_ fixation was calculated by measuring the reduction of acetylene to ethylene and multiplying the ratio of the peak area of ethylene to acetylene by the moles of acetylene added [33]. Free-living ARAs were each normalized by % relative N_2_ fixation compared against 0 mM NH_4_Cl and 1% O_2_ conditions. Rates of fixation for *in-planta* ARAs were calculated between 48 and 72 h by subtracting the mean fraction converted at 48 h from that at 72 h and dividing by the number of hours passed (24 h).

### Colonization assays

Root and rhizosphere colonization assays were performed as described previously [34]. Roots were ground in 10 ml PBS using pestle and mortar to extract firmly associated bacteria. Rhizosphere bacteria were recovered by vortexing the sand–vermiculite substrate with 30 ml PBS. Serial dilutions were plated onto selective TY agar containing nystatin (50 μg ml^−1^), and colony-forming units were calculated.

### Confocal microscopy

GFP-tagged strains were inoculated onto barley seedlings as described for *in-planta* ARA methods. Roots were harvested 7 days post-inoculation, washed in water, sections cut into one cm sections and mounted onto slides with water. Imaging was performed using a ZEISS LSM 880 Axio Imager 2 with a C-Apochromat 40×/1.2 W Korr FCS M27 objective. GFP was excited at 488 nm (3% laser power) and detected using PMT detectors between 493–598 nm [35]. Confocal images were analysed in ZEN 3.6 (blue edition).

### Statistical analysis

All statistical analysis and graphs were produced on Prism (v9.4.1).

## Results and discussion

### Barley root isolation of diazotrophic strains

Microbiota from barley roots were sampled and inoculated into glass universal tubes with 0.175% agar semi-solid minimal media containing different carbon sources and incubated until bacterial growth was observed. Atmospheric N_2_ in the universal head space was the sole source of N. Root-derived mixed bacterial cultures were plated, purified, and screened for *nifH*, revealing 24 colonies. These colonies were inoculated into liquid UMS at 3% O_2_ to examine their N_2_ fixation in free-living culture and eight colonies reduced acetylene (Supplementary Table 5). Colony J2, grown on glucose, had the highest fixation (38.83 nmol ethylene univeral^−1^ hr^−1^), followed by colony B6, isolated and grown on succinate (20.06 nmol ethylene univeral^−1^ hr^−1^). *Azospirillum brasilense* FP2, a known free-living diazotroph used as a positive control, showed 6.48 nmol ethylene univeral^−1^ hr^−1^ in identical conditions. Colonies B6 and J2 were subsequently selected for whole genome sequencing and characterization.

### Genome description

Hybrid Illumina short read and Oxford nanopore long read sequencing was used to assemble B6 and J2 genomes and showed both were similar in size (~5.7 Mb) and architecture, with a single *~*4.4 Mb chromosome and four plasmids between 0.25 and 0.41 Mb (Table 1). Both strains contained a similar number of coding sequences (CDS), 5552 for B6 and 5576 for J2 (Table 1). Assembly of the J2 genome additionally identified a 57 kb contig that could not be assigned to a defined replicon. This contig was flanked by multiple coding sequences annotated as mobilization proteins, including components of a type IV secretion system, suggesting its likely identity as a mobile genetic element.

**Table 1:** Genome statistics of strains B6 and J2.

B6			J2		
Replicon	Size (bp)	GC%	Replicon	Size (bp)	GC%
Chromosome	4 478 470	60.94	Chromosome	4 396 431	61.02
pB6.A	401 832	62.96	pJ2.A	414 512	62.79
pB6.B	301 560	61.39	pJ2.B	325 347	60.42
pB6.C	292 805	60.36	pJ2.C	300 832	61.4
pB6.D	255 576	60.25	pJ2.D	252 799	60.48
			J2 ME^*^	57 005	56.95
Total			Total		
Size (bp)	5 730 243		Size (bp)	5 746 926	
% of genome in plasmids	21.8		% of genome in plasmids	23.5	
% GC	61.04		% GC	61.07	
CDS	5552		CDS	5576	
rRNA operon	4		rRNA operons	4	
tRNA	55		tRNAs	55	

^*^ME = mobile element

**Figure 1:**
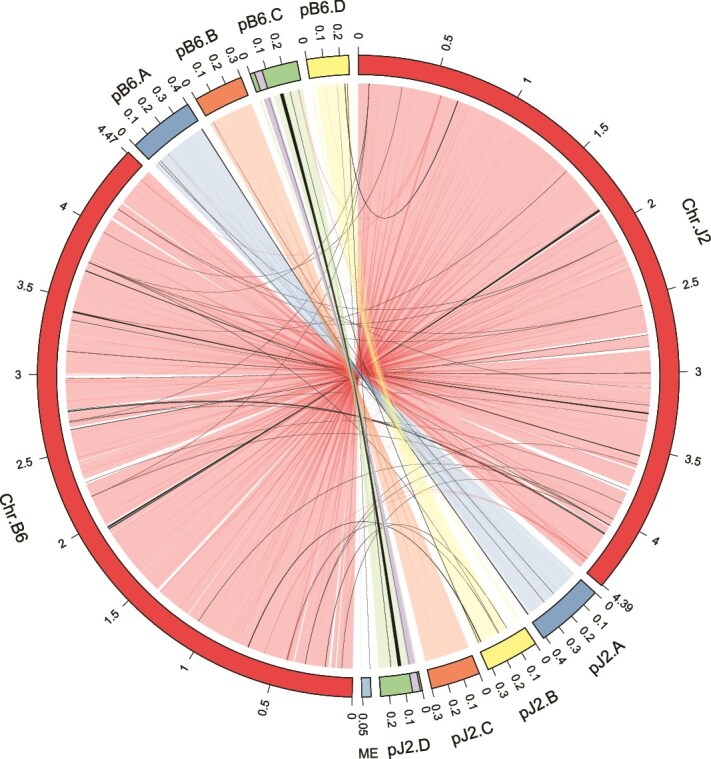
Representation of the genomic comparison between B6 and J2. Chromosome similarities (red) and plasmids with the same colour share the highest synteny. Black lines represent less than 96% similarity in nucleotide identity.

Whole genome synteny analysis between B6 and J2 showed high overall similarity, with a mean nucleotide identity of 97.6% and mean coverage of 94.1% for B6 and 92.7% for J2. Chromosomal comparisons alone showed 96.9% identity, with coverage of 94.7% and 96.4% for B6 and J2, respectively. Each of the strains' four plasmids were named A–D based on size rather than synteny. BLAST analysis of the plasmid replication genes (*repABC*) showed that plasmids with the same *repABC* genes shared the highest synteny, despite differences in size. The most syntenic plasmid pair was pB6.B and pJ2.C, with >98% identity and near complete coverage. The lowest synteny was observed between the *nif–fix* pB6.C and pJ2.D, showing 96% identity and 85.7% coverage (B6) and 99.2% (J2), reflecting differences in size between the two plasmids (0.29 Mb and 0.25 Mb, respectively; Supplementary Table 6, Fig. 1).

Analysis of synteny gaps identified strain-specific regions (>10 kb) unique to either B6 or J2 (Fig. 1, Supplementary Table 7). All exclusive regions contained genes associated with DNA mobilization, including integrases, mobile elements, transposases, or phage-related genes. Some regions encoded genes related to nutrient acquisition, biosynthesis enzymes, and polysaccharide biosynthesis and modification. The J2-specific contig (J2.ME) encoded components of a type IV secretion system, along with phosphonate transporters and enzymes involved in peptidoglycan biosynthesis (Fig. 1, Supplementary Table 7). Overall genome organization supports derivation of B6 and J2 from a common ancestor, followed by independent acquisitions of mobile genetic elements. Notably, B6 contained exclusive regions flanking the *nif–fix* region, suggesting gaining of the N_2_-fixing element at different evolutionary timepoints along with additional gene content. Comparative analysis across strains revealed this *nif–fix* region is conserved within Rhizobiaceae (Fig. 3), consistent with a promiscuous, horizontally transferable N_2_ fixation element.

### Taxonomy of B6 and J2

Predicted proteomes of B6 and J2 were compared against type strains from within Rhizobiaceae and other N_2_-fixing bacteria from Alpha-, Beta-, and Gamma-proteobacteria (Supplementary Fig. 1). AAI analysis identified *Ciceribacter sichuanensis* S101^TS^ as the closest relative of B6 and J2, with AAI values exceeding 98.5% (Table 2). Although *C. sichuanensis* was originally isolated from soybean nodules, it lacks genes for nodulation and cannot induce nodule formation when tested on *Glycine max, Pisum sativum, Vicia faba, Trifolium repens, Vicia angustifolia*, and *Lotus corniculatus* [36]. The highest AAI values observed between B6 and J2 and a genus-defining strain were with *Paenirhizobium daejeonense* L61^T^. *Paenirhizobium* is a recently established and accepted genus that has reshaped the *Ciceribacter* genus, with bacteria formerly known as *Rhizobium daejeonense, C. selenitireducens, C. naphthalenivorans,* now being reassigned as *Paenirhizobium* [37]*.* In light of these changes, *C. sichuanensis* should be reassigned as *Paenirhizobium*; however, the species has not yet been validated for publication by the international code of nomenclature of prokaryotes (ICNP). Strains B6 and J2 share AAI values of 95% and 94.8% AAI (Table 2) and ANI values of 92.7% and 92.84% (Supplementary Table 8) with the genus-defining strain *P. daejeonense* L61^T^, respectively, placing B6 and J2 as members of the *Paenirhizobium* genus but not enough to be assigned to the species *P. daejeonense* [38]. Therefore, in anticipation of taxonomic changes, these strains will be referred to as *Paenirhizobium* sp. B6 and J2. Further validation and deeper taxonomical analysis are required to untangle the taxonomic relationships within Rhizobiaceae (Supplementary Fig. 1).

**Table 2:** AAI values comparing amino acid sequence similarity against other Rhizobiaceae members.

Strains	B6	J2
B6	100	98.76
J2	98.74	100
*Ciceribacter sichuanensis* S101^TS^	98.56	98.54
*Paenirhizobium daejeonense* L61^T^	95.00	94.82
*Rhizobium cremeum* W15^T^	89.83	89.55
*Paenirhizobium terricola* S-51^T^	79.68	79.72
*Paenirhizobium selenitireducens* ATCC BAA-1503^T^	79.13	79.14
*Paenirhizobium naphthalenivorans* NBRC 107585^T^	78.54	78.44
*Ciceribacter lividus* DSM 25528^T*^	75.54	75.64
*Ciceribacter ferrooxidans* F8825^T^	75.42	75.35
*Ciceribacter thiooxidans* F43B^T^	75.19	75.23
*Peteryoungia ipomoeae* shi9-1^T*^	73.35	73.26
*Allorhizobium undicola* ATCC 700741^T*^	71.77	71.70
*Ferirhizobium litorale* KMM 9576^T*^	71.15	70.88
*Agrobacterium tumefaciens* ATCC 4720^T*^	69.88	69.79
*Pseudorhizobium pelagicum* R1-200B4^T*^	69.68	69.62
*Neorhizobium galegae* HAMBI 540^T*^	69.52	69.54
*Rhizobium leguminosarum* USDA 2370^T*^	69.18	68.93


^T^Type strain.

^T*^Type strain that defines the genus.

### Analysis of the N_2_ fixation gene cluster

Genomic analysis identified *nif* and *fix* genes located on plasmids in *Paenirhizobium* sp. B6 (pB6.C) and J2 (pJ2.D). No homologues of nodulation-related genes (*nod, nop, nol*) were found in either strain, indicating they cannot stimulate Nod factor-mediated nodule formation.

**Figure 2:**
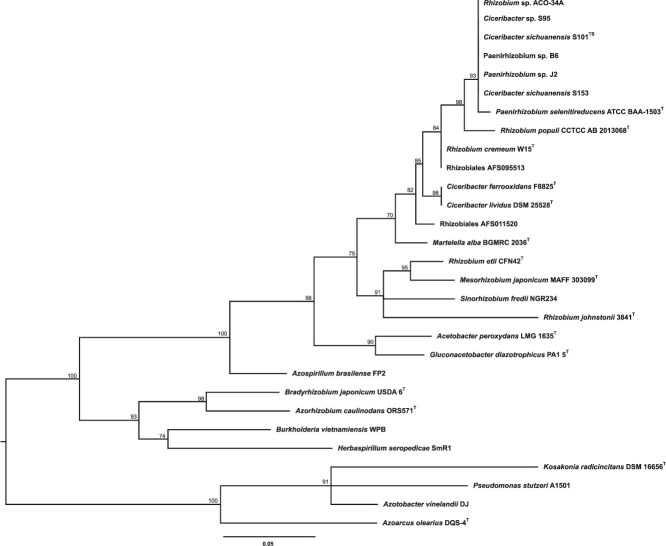
Phylogenetic tree of the NifH protein across free-living and symbiotic proteobacteria.

Phylogenetic analysis of B6 and J2’s NifH placed them within a distinct clade comprising *Paenirhizobium* spp. and several closely related taxa, including *Rhizobium* sp. ACO-34A, *R. populi* CCTCC AB 2013068^T^, *P. cremeum* W15^T^, Rhizobiales AFS095513, Rhizobiales AFS011520, and *Martellela alba* BGMRC 2036^T^ (Fig. 2). Many members of this clade were isolated from root-associated environments, such as *Rhizobium* sp. ACO-A34 and *M. alba* BGMRC 2036^T^ from the rhizospheres of agave and mangrove, respectively [39, 40]. Although some strains are currently classified as *Rhizobium*, phylogenomic analyses indicate closer affiliation with *Paenirhizobium* and *Ciceribacter* (Supplementary Fig. 1).

**Figure 3:**
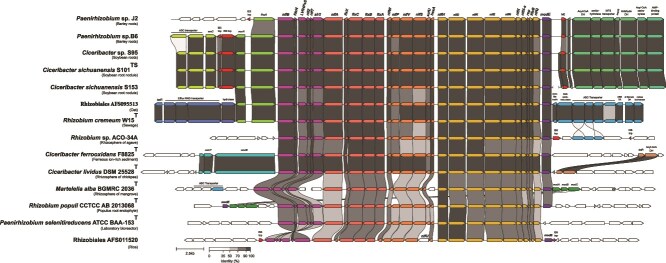
Synteny analysis comparing the genes in *nif-fix* regions (top) and other related genes across other members of Rhizobiaceae.

Comparative analysis of the *nif–fix* regions across this clade revealed a broadly conserved, yet non-canonical, cluster organization (Fig. 3). Most strains contained genes encoding electron transport components (*fdxN, fdxB, fixABCX*), the nitrogenase complex (*nifHDK*), regulatory factors (*nifA, modE*, and *sir2*), FeMo-cofactor biosynthesis (*nifB, nifE, nifN, nifX, nifV, nifQ*), and Fe–S cluster biosynthesis (*nifS, nifU*), together with several accessory genes (*nifW, hesA, nifT, nifP, nifX-associated, rop*). While this architecture retains the modular structure typical of free-living diazotrophs [41], the presence of additional or regulatory elements (e.g. *sir2, modE, rop*) and the separation of the molybdate transport genes, often accompanied by a second, divergent *modE* copy elsewhere in the genome (<40% amino acid identity), indicates a nonclassical, expanded *nif–fix* island likely shaped by lineage-specific regulation or horizontal gene transfer [42, 43].

Despite conservation of internal gene order, genomic context of the *nif–fix* cluster varies among strains. Flanking regions of *Paenirhizobium* sp. B6, *C. sichuanensis* S101^TS^, S153, and S95 were almost identical, whereas J2 shared similarity only downstream of the cluster (Fig. 3). Multiple strains carrying transposable elements adjacent to the *nif–fix* region support the hypothesis that these genes are embedded within or associated with mobile genetic elements. This is consistent with the localization of N_2_ fixation genes on plasmids, integrative conjugative elements (ICEs), or genomic islands in many diazotrophs [44], aligning with whole-genome synteny showing strain-specific flanking regions in J2 (Fig. 1). Conservation of the *nif–fix* context was higher among *C. sichuanensis* strains than in more distantly related taxa such as *P. cremeum* W15^T^ and *Rhizobiales* AFS095513, despite their clustering by cpAAI (Supplementary Table 4).

Several lineage-specific deviations were observed, such as with *M. alba* BGMRC 2036^T^, which lacked *nifP*, encoding a serine acetyltransferase involved in cysteine supply for Fe–S cluster formation. Although non-essential to nitrogenase function, loss of *nifP* is known to reduce nitrogenase activity [45, 46]. A *nifP* mutant in the cyanobacterium *Leptolyngbya boryana* showed a 22% reduction in nitrogenase activity [47], indicating that while *nifP* is not essential, its absence impacts fixation. Except for Rhizobiales AFS011520, all analysed strains contained *sufU* instead of *nifU* upstream of *nifS*. SufU lacks the N-terminal domain characteristic of canonical NifU proteins, suggesting these strains depend on the general Fe–S biogenesis pathway (Isc/Suf/Nfu) for nitrogenase assembly [48]. Although not typically associated with the *nif–fix* cluster, all strains (except *P. selenitireducens* ATCC BAA-1503^T^) harboured a *modE* gene, downstream of *nifQ*, which represses the expression of molybdate transport genes [49]. *P. selenitireducens* ATCC BAA-1503^T^ can reduce selenate to elemental selenium, and since selenate and molybdate are chemically similar oxyanions, this strain may have evolved to constitutively express molybdate/selenate transporters, allowing continuous uptake of both compounds despite the absence of *modE* regulation [50].

### Growth characteristics

*Paenirhizobium* sp. B6 and J2 were isolated on N-free UMS^Succ^ and UMS^Gluc^, respectively, and subsequently tested for growth on common microbiology media. Both strains grew well on TY and LB agar at 28°C. In nutrient-rich liquid media TY, UMS^Gluc^ and UMS^Succ^, B6 and J2 displayed mean generation times (MGTs) typical of fast-growing Alpha-proteobacteria [8, 31, 51]. The shortest MGTs for both strains were observed in UMS^Succ^ after 30 h (B6: 2.59 h ± 0.17 SD; J2: 2.60 h ± 0.14), compared with UMS^Gluc^ (B6: 2.99 h ± 0.12; J2: 2.71 h ± 0.14) and TY (B6: 4.81 h ± 0.10; J2: 3.85 h ± 0.22). UMS^Succ^ was therefore selected for subsequent liquid-culture experiments.

Carbon utilization profiles of both strains were assessed using Biolog PM1 MicroPlates™ and monitored over 50 h (Supplementary Table 9). Of the 95 carbon substrates tested, B6 utilized 46% and J2 75%, indicating broader metabolic versatility in J2. Both strains catabolized several uncommon substrates, including the d–isomer of malic acid. Notably, both grew on single-carbon formic acid, prompting further investigation of their methylotrophic capabilities. Both strains were cultured with UMS agar containing methanol (UMS^MeOH^) with and without lanthanum chloride (LaCl_3_), as pyrroloquinoline quinone–dependent methanol dehydrogenases can be lanthanide-dependent [52]. Growth was observed after 7 days on LaCl_3_-supplemented plates, with poor growth in its absence, indicating B6 and J2 are lanthanide-dependent methylotrophs (Supplementary Fig. 2).

Vitamin auxotrophies were assessed by supplementing UMS^Succ^ individually with thiamine, d-pantothenic acid, or biotin. After 3 days incubation, strain growth was dependent on thiamine but not d-pantothenic acid, or biotin (Supplementary Fig. 2). This thiamine requirement may explain why these strains have not been cultured in the laboratory until recently [36].

### Diazotrophic growth

**Figure 4:**
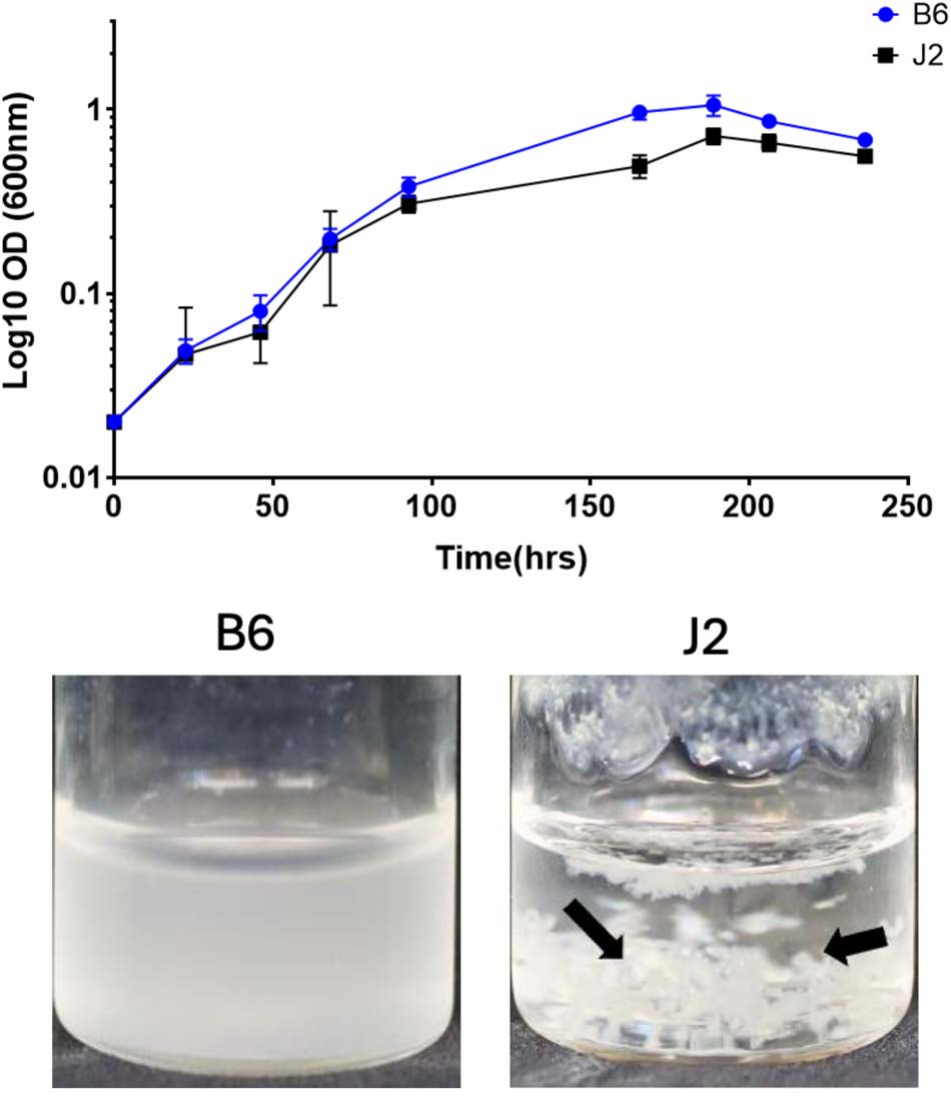
Logarithmic (log_10_) diazotrophic growth curve of B6 (*n* = 4) and J2 (*n* = 4) grown in UMS^Succ^ 1% O_2_ after 10 days. Error bars indicate SD. Panels with images of B6 and J2 in N-free UMS^Succ^ show bacterial aggregates forming in J2 but not B6 cultures in diazotrophic conditions.

The capacity of *Paenirhizobium* sp. B6 and J2 to sustain growth solely by fixing N_2_ in diazotrophic conditions was assessed by cultivation in N-free UMS^Succ^ under microaerobic conditions (1% O_2_) for 10 days. Both strains entered exponential growth after 46–68 h and reached maximal OD after 8 days (Fig. 4). Cultures became increasingly turbid, reflecting slow but sustained growth, with MGTs of 42.6 h ± 2.47 SD and 79.45 h ± 11.63 for B6 and J2 respectively. These results demonstrate both strains can fix atmospheric N_2_ to support growth independent of soluble N, showing they can grow diazotrophically using N_2_ as a sole source of N.

Under N-free conditions, J2 formed visible aggregates whereas B6 did not (Fig. 4). Aggregation and biofilm formation under N limitation has been reported for several free-living diazotrophs and is commonly associated with extracellular polysaccharide production [53, 54]. In *A. brasilense* FP2, N starvation induces aggregation and EPS production, which is believed to reduce intracellular O_2_ tension within cell clusters and thereby protect O_2_-sensitive nitrogenase from degradation [54–56]. Such aggregation may also promote long-term survival under nutrient stress and facilitate persistence in soil environments [54, 57]. Beyond its role in fixation, EPS-mediated aggregation may enhance root attachment and colonization by increasing cell adhesion and biofilm formation on plant root surfaces [58]. The aggregation phenotype observed in J2, but not in B6, also suggests strain-specific strategies for coping with N limitation and O_2_ sensitivity, potentially influencing each strain’s performance in root-associated niches.

### Regulation of N_2_ fixation

**Figure 5:**
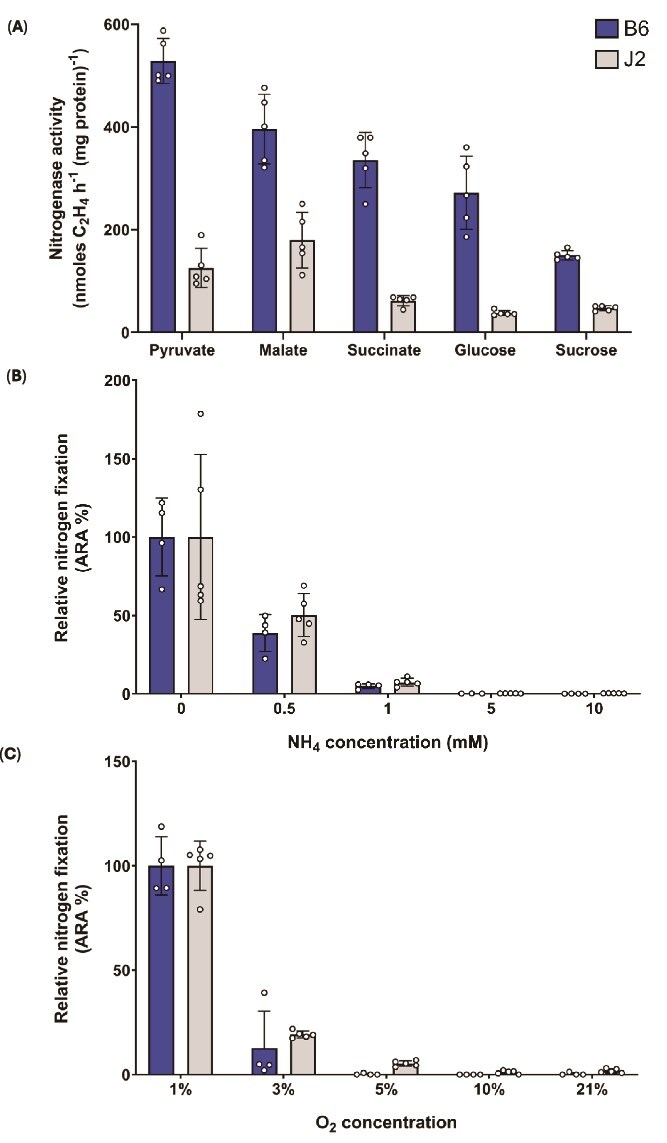
Testing environmental effects on fixation. (A) Free-living fixation of *Paenirhizobium* sp. B6 (blue/dark grey) and J2 (light grey) on sodium pyruvate (30 mM), l-malic acid (20 mM), sodium succinate (20 mM), glucose (10 mM), and sucrose (10 mM) as carbon sources for fixation. Free-living assays were performed in microaerobic conditions (1% O_2_) with gas measurements taken after 24 h. All data were normalized using the final protein content of the culture. Error bars represent SD. (B) Relative levels of N_2_ fixation (ARA %) in free-living conditions with increasing NH_4_^+^ (B) and O_2_ (C) concentrations in B6 and J2 after 16 h growth in UMS^Succ^. Data were normalized by protein (mg) and relative percentage fixation calculated against the total fixation seen in both strains when grown in optimal nitrogen-fixing conditions (0 mM NH_4_^+^ and 1% O_2_). Error bars represent SD.

To characterize environmental regulation of N_2_ fixation in *Paenirhizobium* sp. B6 and J2, the effects of exogenous carbon, ammonium (NH_4_^+^), and O_2_ were examined using free-living ARAs. Both strains fixed N_2_ more efficiently when grown with organic acids rather than sugars (Fig. 5A). In B6, the highest fixation rate was observed with pyruvate (528.24 ± 43.78 SD nmol ethylene^−1^ hr^−1^ (mg protein)^−1^), with the lowest on sucrose (150.16 ± 9.23). Similarly, J2 fixed most efficiently on malate (179.41 ± 54.19) and poorly on glucose (37.56 ± 4.95). Across all substrates, J2 exhibited lower overall nitrogenase activity than B6. Pyruvate, malate, and succinate can be rapidly oxidized to support ATP production and drive N_2_ fixation [59]. In contrast, sucrose and glucose must first be catabolized to pyruvate via the Entner–Doudoroff pathway before entering central energy metabolism, potentially limiting energy availability and reducing fixation efficiency over the 24 h assay period [60, 61]. The marked difference in fixation capacity between B6 and J2, despite highly conserved *nif–fix* clusters (Fig. 3), underscores the influence of broader genomic context and regulatory architecture on diazotrophic performance.

#### Ammonium regulation

Free-living diazotrophs typically favour N assimilation and therefore downregulate nitrogenase expression in the presence of exogenous N [62]. Free-living ARAs were performed across a range of NH_4_^+^ concentrations to assess the impact of NH_4_^+^ on fixation (Fig. 5B). Since experiments were performed in batch cultures and not steady-state conditions, these experiments were limited to 16 h to minimize depletion of low NH_4_^+^ concentrations through bacterial metabolism.

In both strains, N_2_ fixation declined significantly with increasing NH_4_^+^ and was completely inhibited at concentrations ≥5 mM (Fig. 5B). At NH_4_^+^ concentrations up to 0.5 mM, fixation decreased by 61% in B6 and 50% in J2, with a further reduction of 34% and 43% at 1 mM NH_4_^+^, respectively. Complete repression at higher NH_4_^+^ concentrations is consistent with regulation mediated via NifA, the master transcriptional activator of *nif* genes, integrating cellular N status with nitrogenase expression [41]. Sequence analysis of NifA in B6 and J2 revealed strong homology to other O_2_-sensitive NifA proteins from *A. brasilense* (FP2) and *Herbaspirillum seropedicae*, including a conserved cysteine residue motif (Cys-X_11_-Cys-X_19_-Cys-X_4_-Cys) in the linker region between the AAA+ and C-terminal domains [63], suggesting similar redox-sensitive regulation.

Notably, B6 and J2 lack homologues of *nifL* and post-translational regulatory system *draT/draG*, indicating nitrogenase expression in these strains likely relies on regulation by NtrBC or directly by O_2_ [64]. The role of NtrC in free-living N_2_ fixation varies by species, with fixation in *Azotobacter vinelandii, A. brasilense*, and photosynthetic *Bradyrhizobium japonicum* being non-NtrC dependent [65] whereas in *Klebsiella pneumoniae* and *Azorhizobium caulinodans* NtrC is required for efficient *nif* expression [64, 66]. The exact role of NtrBC in *nif* expression in *Paenirhizobium* sp. B6 and J2 requires further elucidation.

#### Oxygen regulation

Unlike symbiotic diazotrophs, fixing N_2_ within O_2_-impermeable nodules, free-living diazotrophs lack physical protection from O_2_ and are sensitive to O_2_-mediated nitrogenase inactivation [67, 68]. To determine the O_2_ tolerance of *Paenirhizobium* sp. B6 and J2 nitrogenase, free-living ARAs were performed with cultures initially equilibrated to 1% O_2_ before being sealed and adjusted to the desired O_2_ concentration (Fig. 5C). Both strains exhibited maximum nitrogenase activity at 1% O_2_, on average fixing at 114.46 ± 16.02 SD nmol ethylene hr^−1^ (mg protein)^−1^ for B6 and 266.27 ± 31.43 for J2. Increasing O_2_ from 1% to 3% resulted in pronounced reductions in fixation by 87% in B6 and 81% in J2. At 5% O_2_, B6 showed near-complete inhibition of nitrogenase activity, whereas J2 low but detectable activity (~5% of that observed at 1% O_2_). A comparable sensitivity to O_2_ has been reported for the free-living diazotroph *Pseudomonas stutzeri* A1501, which also shows sharply reduced N_2_ fixation at O_2_ concentrations above 5% [58].

The partial tolerance to elevated O_2_ observed in J2 may be linked to its aggregating phenotype in fixing conditions. Similar clumping behaviour has been described in other free-living diazotrophs, where EPS-mediated aggregation is thought to protect nitrogenase by creating microoxic environments within cell clusters [54]. Such protection may arise through diffusion barriers imposed by EPS or through elevated respiratory activity within aggregates, collectively reducing local O_2_ tension [53]. These mechanisms are consistent with the maintenance of low-level N_2_ fixation by J2 under otherwise inhibitory O_2_ conditions. Importantly, aggregation did not appear to compromise nitrogenase function in J2 and could enhance diazotrophic resilience in fluctuating O_2_ environments.

### Colonization and N_2_ fixation on barley

**Figure 6:**
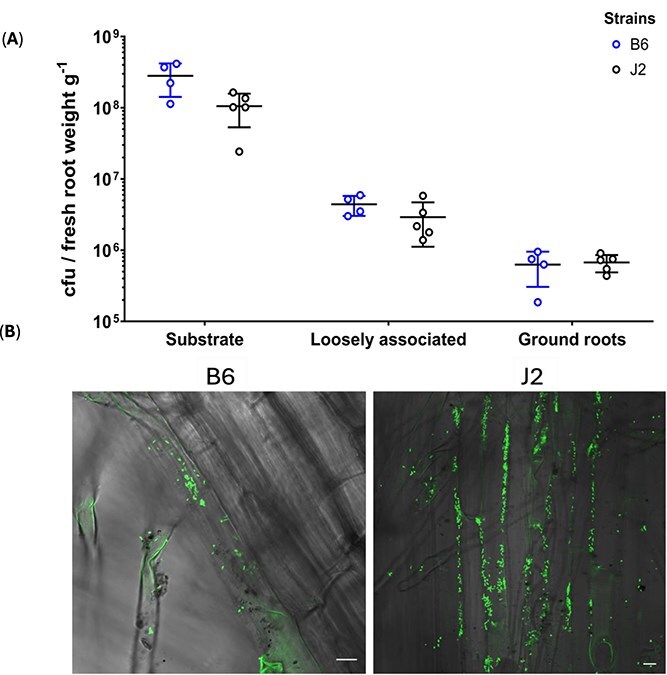
Colonization and confocal visualization of B6 and J2 on barley roots. (A) Quantification of colonization of barley roots by *Paenirhizobium* sp. B6 and J2. B6 (*n* = 4) and J2 (*n* = 5) bacterial colonies counted after extraction from different fractions after 7 days of growth on barley seedlings. Data were log10 transformed before each fraction was analysed using one-way ANOVA and Tukey HSD post hoc test. Error bars represent SD and *P* > .05 not significant (ns). (B) Confocal images of B6-GFP and J2-GFP colonizing the surface of barley roots. Scale bar indicates 10 μm.

Root colonization of *Paenirhizobium* sp. B6 and J2 was quantified before assessing plant-dependent N_2_ fixation. Bacterial numbers on barley roots were determined by colony counts from substrate, loosely associated, and ground root fractions [34]. No significant differences in colonization were observed between the strains in any fraction (Fig. 6A). When total root-associated populations were considered (loosely associated and ground root fractions), B6 and J2 colonized barley roots at comparable densities, reaching 5.03 × 10^6^ CFU g^−1^ fresh root weight (±1.57 × 10^6^ SD) and 3.58 × 10^6^ (±1.95 × 10^6^), respectively. These values fall within the typical range of robust root colonization (10^5^–10^7^ CFU g^−1^ fresh root weight) [69] and are high relative to colonization densities reported for other diazotrophs on pea and barley roots [32]. Together, these data indicate both strains are well adapted for growth in the barley rhizosphere.

Spatial colonization patterns were examined using GFP-tagged versions of B6 and J2 and visualized by confocal microscopy [51]. Imaging of inoculated roots revealed that both strains colonized primarily on the root surface, with no evidence of colonization within the inner cortical tissues, indicating a non-endophytic lifestyle. Representative images showed broad colonization along the root surface, with no apparent preference for specific root zones such as near emerging lateral roots, root hairs, or at the root tip (Fig. 6B). Instead, both strains coalesced along the epidermal root cell margins and occupied surface fissures.

Although B6 and J2 did not colonize deep tissues, a trait often associated with access to plant nutrients, their association with root surface fissures suggests stable colonization. However, no crack-entry was observed on barley roots. Surface-associated colonization nonetheless supports effective interaction with the host and enables these bacteria to potentially contribute fixed N_2_ to the plant.

#### Fixation on barley roots

Since strains B6 and J2 fix N_2_ in free-living conditions, their fixation capabilities on barley roots were tested with plants grown and incubated under microaerobic conditions (1% O_2_), with fixation detected under these conditions being fuelled by carbon from root exudates. *In-planta* ARAs were performed, with gas samples collected 48 and 72 h after injection of 10% acetylene into the container headspace.

**Figure 7:**
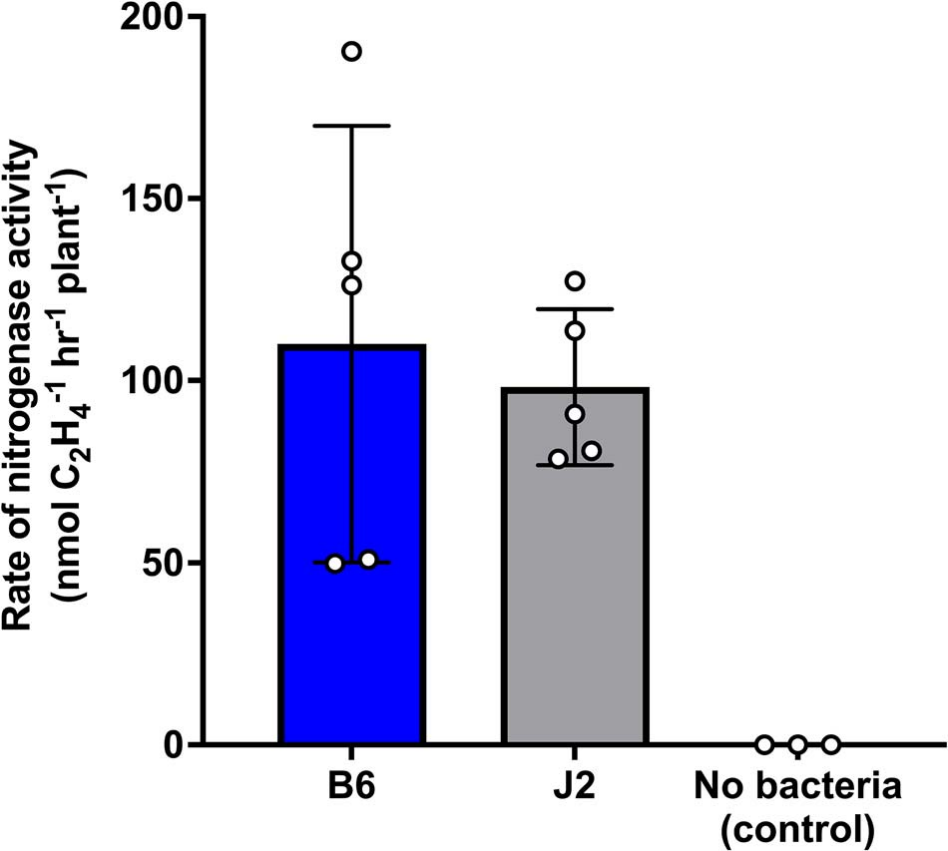
*In-planta* ARAs measuring N_2_-fixation of *Paenirhizobium* sp. B6 (*n* = 5) and J2 (*n* = 5) when in association with barley seedlings inoculated at 0.1 OD_600nm_. Rate of nitrogenase activity calculated between 48 h and 72 h. Error bars represent SD.

Average N_2_ fixation rates, calculated between 48 and 72 h, were 110.04 ± 59.92 nmol ethylene hr^−1^ plant^−1^ for B6 and 98.23 ± 21.38 for J2, with no detectable fixation in mock-inoculated controls (Fig. 7). These data demonstrate that both strains fix substantial amounts of N₂ when growing in association with barley roots under microaerobic conditions, with fixation supported solely by plant root exudates. Notably, fixation rates were similar between B6 and J2 *in-planta*, suggesting the disparity observed under free-living fixing conditions is mitigated in the context of the root environment.

Both strains exhibited nearly double the rates of *in-planta* fixation compared to other well-studied free-living diazotrophs. For example, *Azoarcus olearius* (DQS-4), *P. stutzeri* (A1501), *A. caulinodans* (ORS571), and *A. brasilense* (FP2) fix at rates between 58 and 65 nmol ethylene hr^−1^ plant^−1^ under identical experimental conditions [32]. Many well-studied diazotrophs originate from tropical or subtropical environments and are often not well adapted for colonization of temperate cereal crops. Since B6 and J2 were isolated directly from barley roots, they are more likely better adapted to the barley rhizosphere and therefore more competitive in this environment than non-native diazotrophs.

Root exudate composition varies across plant species and cultivars and is influenced by environmental stresses and plant development [70]. Barley exudates are dominated by organic acids (~50%), with sugars and sugar acids/alcohols (15%), amino acids (11%), and fatty acids (3%) comprising the remainder [71]. This profile is well aligned with the metabolic capabilities of B6 and J2, which fix N_2_ best on organic acids and can catabolize a wide range of carbon sources (Supplementary Table 9), making both strains well suited to free-living fixation in the barley rhizosphere.

## Conclusions

Two strains of *Paenirhizobium* isolated from barley roots were examined and shown to fix high levels of N_2_ in microaerobic conditions when in partnership with plants. Although phylogenetically related to symbiotic rhizobia, both strains lack nodulation genes but do possess canonical *nif–fix* clusters on plasmids, enabling free-living fixation. Their genomes, regulatory architecture, carbon utilization profiles, and general growth reveal them as interesting and tractable systems for studying cereal-associated free-living diazotrophs. Both strains displayed strong colonization and *in-planta* N_2_ fixation, showing they are well adapted for growth in cereal rhizospheres. Notably, strain J2 shows aggregation and biofilm-forming phenotypes that would be beneficial for allowing firm attach to roots. Together, these characteristics position B6 and J2 as experimentally accessible model cereal root diazotrophs for investigating genetic, physiological, and ecological determinants of free-living N_2_ fixation.

## Supplementary Material

Supplementary-material_ycag156

## Data Availability

*Paenirhizobium* sp. B6 and J2 NCBI genomes can be found under BioProject PRJNA1368645 and contains raw reads and assembly data. All other data generated or analysed during this study are included in this published article and its supplementary information files.

## References

[1] Davies-Barnard T, Friedlingstein P. The global distribution of biological nitrogen fixation in terrestrial natural ecosystems. *Global Biogeochem Cycles* 2020;34:e2019GB00638. 10.1029/2019GB006387

[2] Davies-Barnard T, Zaehle S, Friedlingstein P. Assessment of the impacts of biological nitrogen fixation structural uncertainty in CMIP6 earth system models. *Biogeosciences* 2022;19:3491–503. 10.5194/bg-19-3491-2022

[3] Reed S, Cleveland CC, Townsend AR. Functional biology of heterotrophic nitrogen-fixing bacteria. *Annu Rev Ecol Evol Syst* 2011;42:489–512. 10.1146/annurev-ecolsys-102710-145034

[4] Geddes BA, Ryu MH, Mus F., et al. Use of plant colonizing bacteria as chassis for transfer of N_2_-fixation to cereals. *Curr Opin Biotechnol* 2015;32:216–22. 10.1016/j.copbio.2015.01.004 25626166

[5] Khan S, Nadir S, Iqbal S., et al. Towards a comprehensive understanding of free-living nitrogen fixation. *Circ Agric Syst* 2021;1:1–11. 10.48130/cas-2021-0013

[6] Mirza BS, Rodrigues JLM. Development of a direct isolation procedure for free-living diazotrophs under controlled hypoxic conditions. *Appl Environ Microbiol* 2012;78:5542–9. 10.1128/AEM.00714-12 22660701 PMC3406155

[7] Baldani JI, Reis VM, Videira SS., et al. The art of isolating nitrogen-fixing bacteria from non-leguminous plants using N-free semi-solid media: a practical guide for microbiologists. Plant and Soil 2014;384:413–31. 10.1007/sl

[8] Wheatley RM, Ramachandran VK, Geddes BA., et al. Role of O_2_ in the growth of *Rhizobium leguminosarum* bv. *viciae* 3841 on glucose and succinate. *J Bacteriol* 2017;199:e00572–16. 10.1128/JB.00572-16 27795326 PMC5165102

[9] Prell J, Bourdès A, Kumar S., et al. Role of symbiotic auxotrophy in the rhizobium-legume symbioses. *PloS One* 2010;5:e13933. 10.1371/journal.pone.0013933 21085630 PMC2978685

[10] Tkacz A, Bestion E, Bo Z., et al. Influence of plant fraction, soil, and plant species on microbiota: a multikingdom comparison. *mBio* 2020;11:e02785–19. 10.1128/mBio.02785-19 32019791 PMC7002342

[11] Attia S, Russel J, Mortensen MS., et al. Unexpected diversity among small-scale sample replicates of defined plant root compartments. *ISME J* 2022;16:997–1003. 10.1038/s41396-021-01094-7 34759302 PMC8940884

[12] Beringer JE . R factor transfer in *rhizobium leguminosarum*. *Microbiology (N Y)* 1974;84:188–98. 10.1099/00221287-84-1-188 4612098

[13] Bertani G . Studies on lysogenesis. I. The mode of phage liberation by lysogenic *Escherichia coli*. *J Bacteriol* 1951;62:293–300. 10.1128/jb.62.3.293-300.1951 14888646 PMC386127

[14] Poole PS, Schofiel NA, Reid CJ., et al. Identification of chromosomal genes located downstream of DctD that affect the requirement for calcium and the lipopolysaccharide layer of *Rhizobium leguminosarum*. *Microbiology (N Y)* 1994;140 (Pt 10):2797–809. 10.1099/00221287-140-10-2797 8000544

[15] Tkacz A, Ledermann R, Martyn A., et al. Nodulation and nitrogen fixation in *Medicago Truncatula* strongly alters the abundance of its root microbiota and subtly affects its structure. *Environ Microbiol* 2022;24:5524–33. 10.1111/1462-2920.16164 36054464 PMC9804836

[16] Angel R, Nepel M, Panhölzl C., et al. Evaluation of primers targeting the diazotroph functional gene and development of NifMAP - a bioinformatics pipeline for analysing NifH amplicon data. *Front Microbiol* 2018;9:703. 10.3389/fmicb.2018.00703 PMC5936773 29760683

[17] Antipov D, Korobeynikov A, McLean JS., et al. HybridSPAdea: an algorithm for hybrid assembly of short and long reads. *Bioinformatics* 2016;32:1009–15. 10.1093/bioinformatics/btv688 26589280 PMC4907386

[18] Wick RR, Judd LM, Gorrie CL., et al. Unicycler: resolving bacterial genome assemblies from short and long sequencing reads. *PLoS Comput Biol* 2017;13:e1005595. 10.1371/journal.pcbi.1005595 28594827 PMC5481147

[19] Brettin T, Davis JJ, Disz T., et al. RASTtk: a modular and extensible implementation of the RAST algorithm for building custom annotation pipelines and annotating batches of genomes. *Sci Rep* 2015;5:8365. 10.1038/srep08365 25666585 PMC4322359

[20] Kurtz S, Phillippy A, Delcher AL., et al. Versatile and open software for comparing large genomes. *Genome Biol* 2004;5:R12. 10.1186/gb-2004-5-2-r12 14759262 PMC395750

[21] Krzywinski M, Schein J, Birol İ., et al. Circos: an information aesthetic for comparative genomics. *Genome Res* 2009;19:1639–45. 10.1101/gr.092759.109 19541911 PMC2752132

[22] Kim D, Park S, Chun J. Introducing EzAAI: a pipeline for high throughput calculations of prokaryotic average amino acid identity. *J Microbiol* 2021;59:476–80. 10.1007/s12275-021-1154-0 33907973

[23] Kuzmanović N, Fagorzi C, Mengoni A., et al. Taxonomy of rhizobiaceae revisited: proposal of a new framework for genus delimitation. *Int J Syst Evol Microbiol* 2022;72:005243. 10.1099/ijsem.0.005243 PMC9558580 35238735

[24] Jain C, Rodriguez-R LM, Phillippy AM., et al. High throughput ANI analysis of 90K prokaryotic genomes reveals clear species boundaries. *Nat Commun* 2018;9:5114. 10.1038/s41467-018-07641-9 30504855 PMC6269478

[25] Kumar S, Stecher G, Li M., et al. MEGA X: molecular evolutionary genetics analysis across computing platforms. *Mol Biol Evol* 2018;35:1547–9. 10.1093/molbev/msy096 29722887 PMC5967553

[26] Sievers F, Higgins DG. Clustal omega for making accurate alignments of many protein sequences. *Protein Sci* 2018;27:135–45. 10.1002/pro.3290 28884485 PMC5734385

[27] Minh BQ, Schmidt HA, Chernomor O., et al. Corrigendum to: IQ-TREE 2: new models and efficient methods for phylogenetic inference in the genomic era. *Mol Biol Evol* 2020;37:2461–1. 10.1093/molbev/msaa131 32556291 PMC7403609

[28] Junier T, Zdobnov EM. The Newick utilities: high-throughput phylogenetic tree processing in the UNIX Shell. *Bioinformatics* 2010;26:1669–70. 10.1093/bioinformatics/btq243 20472542 PMC2887050

[29] van den Belt M, Gilchrist C, Booth TJ., et al. CAGECAT: the CompArative GEne cluster analysis toolbox for rapid search and visualisation of homologous gene clusters. *BMC Bioinformatics* 2023;24:181. 10.1186/s12859-023-05311-2 37131131 PMC10155394

[30] Ditta G, Stanfield S, Corbin D., et al. Broad host range DNA cloning system for gram-negative bacteria: construction of a gene bank of *Rhizobium meliloti*. *Proc Natl Acad Sci* 1980;77:7347–51. 10.1073/pnas.77.12.7347 7012838 PMC350500

[31] Haskett TL, Karunakaran R, Batista MB., et al. Control of nitrogen fixation and ammonia excretion in *Azorhizobium caulinodans*. *PLoS Genet* 2022;18:e1010276–20. 10.1371/journal.pgen.1010276 35727841 PMC9249168

[32] Haskett TL, Knights HE, Jorrin B., et al. A simple *in situ* assay to assess plant-associative bacterial nitrogenase activity. *Front Microbiol* 2021;12:690439. 10.3389/fmicb.2021.690439 34248916 PMC8261070

[33] Herridge DF, Giller KE. Measurement of nitrogen fixation. In: Howieson J.G., Dilworth M.J. (eds.), Working with Rhizobia. Canberra: Australian Centre for International Agriculture Research, 2016, 187–218 ISBN 978 1 925436 18 1 (PDF).

[34] Wheatley RM, Ford BL, Li L., et al. Lifestyle adaptations of rhizobium from rhizosphere to symbiosis. *Proc Natl Acad Sci* 2020;117:23823–34. 10.1073/pnas.2009094117 32900931 PMC7519234

[35] Haskett TL, Geddes BA, Paramasivan P., et al. Rhizopine biosensors for plant-dependent control of bacterial gene expression. *Environ Microbiol* 2022;25:383–96. 10.1111/1462-2920.16288 36428208 PMC10107442

[36] Zhang Y, Chen Y, Penttinen P., et al. *Ciceribacter sichuanensis* Sp. Nov., a plant growth promoting Rhizobacterium isolated from root nodules of soybean in Sichuan, China. *Antonie Van Leeuwenhoek* 2024;117:46. 10.1007/s10482-024-01941-5 38427093

[37] Ma T, Xue H, Piao C., et al. Phylogenomic reappraisal of the family Rhizobiaceae at the genus and species levels, including the description of *Ectorhizobium quercum* gen. Nov., Sp. Nov. *Front Microbiol* 2023;14:1207256. 10.3389/fmicb.2023.1207256 37601364 PMC10434624

[38] diCenzo GC, Yang Y, Young JPW., et al. Refining the taxonomy of the order Hyphomicrobiales (Rhizobiales) based on whole genome comparisons of over 130 type strains. *Int J Syst Evol Microbiol* 2024;74:006328. 10.1099/ijsem.0.006328 PMC11092082 38619983

[39] Ruíz-Valdiviezo VM, Rogel-Hernandez MA, Guerrero G., et al. Complete genome sequence of a novel non-nodulating rhizobium species isolated from *Agave americana* L. Rhizosphere. *Genome Announc* 2017;5:e01280–17. 10.1128/genomeA.01280-17 PMC5690337 29146860

[40] Yang E, Liu J, Chen D., et al. *Rhizobium cremeum* Sp. Nov., isolated from sewage and capable of acquisition of heavy metal and aromatic compounds resistance genes. *Syst Appl Microbiol* 2022;45:126322. 10.1016/j.syapm.2022.126322 35427953

[41] Dixon R, Kahn D. Genetic regulation of biological nitrogen fixation. *Nat Rev Microbiol* 2004;2:621–31. 10.1038/nrmicro954 15263897

[42] Tao J, Wang S, Liao T., et al. Evolutionary origin and ecological implication of a unique *nif* island in free-living Bradyrhizobium lineages. *ISME J* 2021;15:3195–206. 10.1038/s41396-021-01002-z 33990706 PMC8528876

[43] Grunden AM, Shanmugam KT. Molybdate transport and regulation in bacteria. *Arch Microbiol* 1997;168:345–54. 10.1007/s002030050508 9325422

[44] Wardell GE, Hynes MF, Young PJ., et al. Why are Rhizobial symbiosis genes mobile? *Philos Trans R Soc B Biol Sci* 2022;377:20200471. 10.1098/rstb.2020.0471 PMC8628070 34839705

[45] Evans DJ, Jones R, Woodley PR., et al. Nucleotide sequence and genetic analysis of the *Azotobacter chroococcum nifUSVWZM* gene cluster, including a new gene (*nifP*) which encodes a serine acetyltransferase. *J Bacteriol* 1991;173:5457–69. 10.1128/jb.173.17.5457-5469.1991 1885524 PMC208258

[46] Fujita Y, Uesaka K. Nitrogen fixation in cyanobacteria. In: Kageyama H., Waditee-Sirisattha E. (eds.), Cyanobacterial Physiology. Elsevier, 2022, 29–45 10.1016/B978-0-323-96106-6.00007-1.

[47] Nonaka A, Yamamoto H, Kamiya N., et al. Accessory proteins of the nitrogenase assembly, NifW, NifX/NafY, and NifZ, are essential for diazotrophic growth in the non-heterocystous cyanobacterium *Leptolyngbya boryana*. *Front Microbiol* 2019;10:495. 10.3389/fmicb.2019.00495 30930880 PMC6428710

[48] Johnson DC, Dean DR, Smith AD., et al. Structure, function, and formation of biological iron-sulphur clusters. *Annu Rev Biochem* 2005;74:247–81. 10.1146/annurev.biochem.74.082803.133518 15952888

[49] Grunden AM, Ray RM, Rosentel JK., et al. Repression of the *Escherichia coli modABCD* (Molybdate transport) operon by ModE. *J Bacteriol* 1996;178:735–44. 10.1128/jb.178.3.735-744.1996 8550508 PMC177720

[50] Hunter WJ . A *Rhizobium selenitireducens* protein showing selenite reductase activity. *Curr Microbiol* 2014;68:311–6. 10.1007/s00284-013-0474-7 24474405

[51] Jorrin B, Haskett TL, Knights HE., et al. Stable, fluorescent markers for tracking synthetic communities and assembly dynamics. *Microbiome* 2024;12:81. 10.1186/s40168-024-01792-2 38715147 PMC11075435

[52] Wegner C-E, Gorniak L, Riedel S., et al. Lanthanide-dependent methylotrophs of the family Beijerinckiaceae: physiological and genomic insights. *Appl Environ Microbiol* 2019;86:e01830–19. 10.1128/AEM.01830-19 31604774 PMC6912076

[53] Wang D, Xu A, Elmerich C., et al. Biofilm formation enables free-living nitrogen-fixing Rhizobacteria to fix nitrogen under aerobic conditions. *ISME J* 2017;11:1602–13. 10.1038/ismej.2017.30 28338674 PMC5520150

[54] Bible AN, Khalsa-Moyers GK, Mukherjee T., et al. Metabolic adaptations of *Azospirillum brasilense* to oxygen stress by cell-to-cell clumping and flocculation. *Appl Environ Microbiol* 2015;81:8346–57. 10.1128/AEM.02782-15 26407887 PMC4644645

[55] Bible AN, Stephens BB, Ortega DR., et al. Function of a chemotaxis-like signal transduction pathway in modulating motility, cell clumping, and cell length in the Alphaproteobacterium *Azospirillum brasilense*. *J Bacteriol* 2008;190:6365–75. 10.1128/JB.00734-08 18641130 PMC2565992

[56] Bible A, Russell MH, Alexandre G. The *Azospirillum brasilense* Che1 chemotaxis pathway controls swimming velocity, which affects transient cell-to-cell clumping. *J Bacteriol* 2012;194:3343–55. 10.1128/JB.00310-12 22522896 PMC3434747

[57] Huerta JM, Aguilar I, López-Pliego L., et al. The role of the NcRNA RgsA in the oxidative stress response and biofilm formation in Azotobacter Vinelandii. *Curr Microbiol* 2016;72:671–9. 10.1007/s00284-016-1003-2 26858204

[58] Meneses CHSG, Rouws LFM, Simões-Araújo JL., et al. Exopolysaccharide production is required for biofilm formation and plant colonization by the nitrogen-fixing endophyte *Gluconacetobacter diazotrophicus*. *Mol Plant Microbe Interact* 2011;24:1448–58. 10.1094/MPMI-05-11-0127 21809982

[59] Schulte CCM, Borah K, Wheatley RM., et al. Metabolic control of nitrogen fixation in rhizobium-legume symbioses. *Sci Adv* 2021;7:eabh2433. 10.1126/sciadv.abh2433 PMC8324050 34330708

[60] Araujo WL, Nunes-Nesi A, Nikoloski Z., et al. Metabolic control and regulation of the tricarboxylic acid cycle in photosynthetic and heterotrophic plant tissues. *Plant Cell Environ* 2012;35:1–21. 10.1111/j.1365-3040.2011.02332.x 21477125

[61] Alleman AB, Mus F, Peters JW. Metabolic model of the nitrogen-fixing obligate aerobe *Azotobacter vinelandii* predicts its adaptation to oxygen concentration and metal availability. *mBio* 2021;12:e02593–21. 10.1128/mBio.02593-21 34903060 PMC8686835

[62] Batista MB, Dixon R. Manipulating nitrogen regulation in diazotrophic bacteria for agronomic benefit. *Biochem Soc Trans* 2019;47:603–14. 10.1042/BST20180342 30936245 PMC6490700

[63] Oliveira MAS, Baura VA, Aquino B., et al. Role of conserved cysteine residues in *Herbaspirillum seropedicae* NifA activity. *Res Microbiol* 2009;160:389–95. 10.1016/j.resmic.2009.06.002 19573596

[64] Merrick MJ . Nitrogen control of the *nif* regulon in *Klebsiella pneumoniae*: involvement of the NtrA gene and analogies between NtrC and NifA. *EMBO J* 1983;2:39–44. 10.1002/j.1460-2075.1983.tb01377.x 11894906 PMC555083

[65] Yang Z, Li Q, Yan Y., et al. Master regulator NtrC controls the utilization of alternative nitrogen sources in *Pseudomonas stutzeri* A1501. *World J Microbiol Biotechnol* 2021;37:177. 10.1007/s11274-021-03144-w 34524580 PMC8443478

[66] Pawlowski K, Klosse U, de Bruijn FJ. Characterization of a novel *Azorhizobium caulinodans* ORS571 two-component regulatory system, NtrY/NtrX, involved in nitrogen fixation and metabolism. *Mol Gen Genet* 1991;231:124–38. 10.1007/BF00293830 1661370

[67] Ledermann R, Schulte CCM, Poole PS. How rhizobia adapt to the nodule environment. *J Bacteriol* 2021;203:e0053920. 10.1128/JB.00539-20 33526611 PMC8315917

[68] Tang Y, Qin D, Tian Z., et al. Diurnal switches in diazotrophic lifestyle increase nitrogen contribution to cereals. *Nat Commun* 2023;14:7516. 10.1038/s41467-023-43370-4 37980355 PMC10657418

[69] Bulgarelli D, Schlaeppi K, Spaepen S., et al. Structure and functions of the bacterial microbiota of plants. *Annu Rev Plant Biol* 2013;64:807–38. 10.1146/annurev-arplant-050312-120106 23373698

[70] McLaughlin S, Zhalnina K, Kosina S., et al. The core metabolome and root exudation dynamics of three phylogenetically distinct plant species. *Nat Commun* 2023;14:1649. 10.1038/s41467-023-37164-x 36964135 PMC10039077

[71] Naveed M, Brown LK, Raffan AC., et al. Plant exudates may stabilize or weaken soil depending on species, origin and time. *Eur J Soil Sci* 2017;68:806–16. 10.1111/ejss.12487 29263712 PMC5726377

