# Supplementary material for: Non-nodulating Paenirhizobium fix nitrogen in association with cereal roots
Source: ISME Commun. 2026 Jun 24;6(1):ycag156. doi: 10.1093/ismeco/ycag156 (PMC13455631; doi:10.1093/ismeco/ycag156)
Supplement: Supplementary file 1 [file supplementary-material_ycag156.zip › Supplementary material.docx]

>Rhizobium sp. ACO-34A

MAALRQIAFYGKGGIGKSTTSQNTLAALVDLGQKILIVGCDPKADSTRLILNSKAQDTVLSLAAQEGSVEDLELEDVLKIGYKGIKCVESGGPEPGVGCAGRGVITSINFLEENGAYDDVDYVSYDVLGDVVCGGFAMPIRENKAQEIYIVMSGEMMALYAANNIAKGILKYANSGGVRLGGLICNERQTDRELDLAEALSARLNSKLIHFVPRDNIVQHAELRKQTVIEYAPNSQQAQEYRQLAQKIHDNSGKGTIPTPITMEELEDMLLAFGIMKTDEQMLAELAAKEAAKQAVAAV

>Ciceribacter sp. S95

MAALRQIAFYGKGGIGKSTTSQNTLAALVDLGQKILIVGCDPKADSTRLILNSKAQDTVLSLAAQEGSVEDLELEDVLKIGYKGIKCVESGGPEPGVGCAGRGVITSINFLEENGAYDDVDYVSYDVLGDVVCGGFAMPIRENKAQEIYIVMSGEMMALYAANNIAKGILKYANSGGVRLGGLICNERQTDRELDLAEALSARLNSKLIHFVPRDNIVQHAELRKQTVIEYAPNSQQAQEYRQLAQKIHDNSGKGTIPTPITMEELEDMLLAFGIMKTDEQMLAELAAKEAAKQAVAAV

>Acetobacter peroxydans LMG 1635

MGKLRQIAFYGKGGIGKSTTSQNTLAALVEMGQKILIVGCDPKADSTRLILNAKAQDTVLSLAAEAGSVEDLELEDVLKIGYKGIKCVESGGPEPGVGCAGRGVITSINFLEENGAYDDVDYVSYDVLGDVVCGGFAMPIRENKAQEIYIVMSGEMMALYAANNIAKGILKYAHSGGVRLGGLICNERQTDREYDLADALAKRLNSKLVHFVPRDNIVQHAELRRQTVIEYAPDSQQAAEYRELARKVHENSGKGTVPTPITMEELEDMLLEFGIMKTEEQQLAELAAKEAAKAAMA

>Sinorhizobium fredii NGR234

MAGLRQIAFYGKGGIGKSTTSQNTLAALVDLGQKILIVGCDPKADSTRLILNAKAQDTVLHLAAKEGSVEDLEVEDVLKVGYKGIKCVESGGPEPGVGCAGRGVITSINFLEENGAYDDVDYVSYDVLGDVVCGGFAMPIRENKAQEIYIVMSGEMMALYAANNIAKGILKYAHSGGVRLGGLICNERQTDRELDLAEALAAKLNSRLIHFVPRDNIVQHAELRKMTVIQYAPESQQAAEYRALADKIHANSGQGTVPTPITMEELEDMLLDFGVMKTDEQMLAELQAKEAAAAAQ

>Rhizobium etli CFN42

MSDLRQIAFYGKGGIGKSTTSQNTLAALVDLGQKILIVGCDPKADSTRLILNAKAQDTVLHLAAQEGSVEDLELEDVLKAGYKGIKCVESGGPEPGVGCAGRGVITSINFLEENGAYDDVDYVSYDVLGDVVCGGFAMPIRENKAQEIYIVMSGEMMALYAANNIAKGILKYAHSGGVRLGGLICNERQTDRELDLSEALAARLNSKLIHFVPRDNIVQHAELRKMTVIQYAPDSKQAGEYRALAEKIHANSGQGTIPTPITMEELEDMLLDFGIMKSDEQMLAELQAKESAVVAAQ

>Ciceribacter sp B6

MAALRQIAFYGKGGIGKSTTSQNTLAALVDLGQKILIVGCDPKADSTRLILNSKAQDTVLSLAAQEGSVEDLELEDVLKIGYKGIKCVESGGPEPGVGCAGRGVITSINFLEENGAYDDVDYVSYDVLGDVVCGGFAMPIRENKAQEIYIVMSGEMMALYAANNIAKGILKYANSGGVRLGGLICNERQTDRELDLAEALSARLNSKLIHFVPRDNIVQHAELRKQTVIEYAPNSQQAQEYRQLAQKIHDNSGKGTIPTPITMEELEDMLLAFGIMKTDEQMLAELAAKEAAKQAVAAV

>Ciceribacter sp J2

MAALRQIAFYGKGGIGKSTTSQNTLAALVDLGQKILIVGCDPKADSTRLILNSKAQDTVLSLAAQEGSVEDLELEDVLKIGYKGIKCVESGGPEPGVGCAGRGVITSINFLEENGAYDDVDYVSYDVLGDVVCGGFAMPIRENKAQEIYIVMSGEMMALYAANNIAKGILKYANSGGVRLGGLICNERQTDRELDLAEALSARLNSKLIHFVPRDNIVQHAELRKQTVIEYAPNSQQAQEYRQLAQKIHDNSGKGTIPTPITMEELEDMLLAFGIMKTDEQMLAELAAKEAAKQAVAAV

>Ciceribacter selenitireducens ATCC BAA-1503

MAALRQIAFYGKGGIGKSTTSQNTLAALVDLGQKILIVGCDPKADSTRLILNSKAQDTVLSLAAQEGSVEDLELEDVLKIGYKGIKCVESGGPEPGVGCAGRGVITSINFLEENGAYDDVDYVSYDVLGDVVCGGFAMPIRENKAQEIYIVMSGEMMALYAANNIAKGILKYANSGGVRLGGLICNERQTDRELDLAEALSARLNSKLIHFVPRDNIVQHAELRKQTVIEYAPNSQQADEYRQLAQKIHNNSGKGTIPTPITMEELEDMLLAFGIMKTDEQMLAELAAKEAAKQAVAAV

>Ciceribacter ferrooxidans F8825

MSGLRQIAFYGKGGIGKSTTSQNTLAALVDLGQKILIVGCDPKADSTRLILNSKAQDTVLSLAAQEGSVEDLELEDVLKIGYKGIKCVESGGPEPGVGCAGRGVITSINFLEENGAYDDVDYVSYDVLGDVVCGGFAMPIRENKAQEIYIVMSGEMMALYAANNIAKGILKYANSGGVRLGGLICNERQTDRELDLAEALAARLNSKLIHFVPRDNIVQHAELRKQTVIEYAPNSQQAQEYRQLAEKIHNNSGKGTIPTPITMEELEDMLLAFGIMKTDEQMLAELQAKEASAAAH

>Ciceribacter lividus DSM 25528

MSGLRQIAFYGKGGIGKSTTSQNTLAALVDLGQKILIVGCDPKADSTRLILNSKAQDTVLSLAAQEGSVEDLELEDVLKIGYKGIKCVESGGPEPGVGCAGRGVITSINFLEENGAYDDVDYVSYDVLGDVVCGGFAMPIRENKAQEIYIVMSGEMMALYAANNIAKGILKYANSGGVRLGGLICNERQTDRELDLAEALAARLNSKLIHFVPRDNIVQHAELRKQTVIEYAPNSQQAQEYRQLAEKIHNNSGKGTIPTPITMEELEDMLLAFGIMKTDEQMLAELQAKEASAAAH

>Rhizobium cremeum W15

MTALRQIAFYGKGGIGKSTTSQNTLAALVDLGQKILIVGCDPKADSTRLILNSKAQDTVLSLAAQEGSVEDLELEDVLKIGYKGIKCVESGGPEPGVGCAGRGVITSINFLEENGAYDDVDYVSYDVLGDVVCGGFAMPIRENKAQEIYIVMSGEMMALYAANNIAKGILKYANSGGVRLGGLICNERQTDRELDLAEALAARLNSKLIHFVPRDNIVQHAELRKQTVIEYAPNSQQAQEYRQLAQKIHDNSGKGTIPTPITMEELEDMLLAFGIMKTDEQMLAELAAKEAAAAAH

>Rhizobium johnstonii 3841

MAALRQIAFYGKGGIGKSTTSQNTLAALVDHGQKILIVGCDPKADSTRLILNSKAQDTVLDLAATRGSVEDLELEDVLKVGYKGIKCVESGGPEPGVGCAGRGVITSINFLEENGAYNDVDYVSYDVLGDVVCGGFAMPIRENKAQEIYIVMSGEMMALYAANNIARGILKYAAGGSVRLGGLICNERQTDRELDLAEALAAKLNSKLIHFVPRDNIVQHAELRKMTVIQYAPDSQQAAEYRTLAQRIHDNSGKGTIPTPITMEELEDMLLDFGIMKTDEQMLAELQARDAKLKAAQ

>Bradyrhizobium japonicum USDA 6

MASLRQIAFYGKGGIGKSTTSQNTLAALAEMGQKILIVGCDPKADSTRLILHAKAQDTILSLAASAGSVEDLELEDVMKVGYQDIRCVESGGPEPGVGCAGRGVITSINFLEENGAYENIDYVSYDVLGDVVCGGFAMPIRENKAQEIYIVMSGEMMAMYAANNISKGILKYANSGGVRLGGLICNERQTDKELELAEALAKKLGTQLIYFVPRDNVVQHAELRRMTVLEYAPDSKQADHYRKLAAKVHNNGGKGIIPTPISMDELEDMLMEHGIIKAVDESIIGKTAAELAAS

>Azospirillum brasilense FP2

MSLRQIAFYGKGGIGKSTTSQNTLAALVELDQKILIVGCDPKADSTRLILHAKAQDTVLHLAAEAGSVEDLELEDVLKIGYKGIKCVESGGPEPGVGCAGRGVITSINFLEENGAYDDVDYVSYDVLGDVVCGGFAMPIRENKAQEIYIVMSGEMMALYAANNIAKGILKYAHSGGVRLGGLICNERQTDKEIDLASALAARLGTQLIHFVPRDNIVQHAELRRMTVIEYAPDSQQAQEYRQLANKVHANKGKGTIPTPITMEELEEMLMDFGIMKSEEQQLAELQAKEAAKA

>Kosakonia radicincitans DSM 16656

MTMRQCAIYGKGGIGKSTTTQNLVAALAEMGKKVMIVGCDPKADSTRLILHAKAQNTIMEMAAEVGSVEDLELEDVLQIGYGDVRCAESGGPEPGVGCAGRGVITAINFLEEEGAYVPDLDFVFYDVLGDVVCGGFAMPIRENKAQEIYIVCSGEMMAMYAANNISKGIVKYAKSGKVRLGGLICNSRQTDREDELIMALAEKLGTQMIHFVPRDNIVQRAEIRRMTVIEYDPKCNQANEYRSLANKIVNNTKMVVPTPITMDELEELLMEFGIMDVEDASIIGKTAAEENAV

>Pseudomonas stutzeri A1501

MAMRQCAIYGKGGIGKSTTTQNLVAALAELGKKVMIVGCDPKADSTRLILHSKAQNTIMEMAAEAGTVEDLELEDVLKTGYGDIKCVESGGPEPGVGCAGRGVITAINFLEEEGAYEDDLDFVFYDVLGDVVCGGFAMPIRENKAQEIYVVCSGEMMAMYAANNICKGIVKYANSGSVRLGGLICNSRNTDREDELIMALADKLGSQMIHFVPRDNVVQRAEIRRMTVIEYDPAAKQADEYRTLAKKIVENKKLVIPTPISMDELEALLMEFGIMDEEDMTIVGKTAAEEVVA

>Burkholderia vietnamiensis WPB

MSQLRQIAFYGKGGIGKSTTSQNTLAALTDLGQKILIVGCDPKADSTRLILHAKAQDTILSLAAEAGSVEDLELEDVMKIGYKDIRCVESGGPEPGVGCAGRGVITSINFLEENGAYDGVDYVSYDVLGDVVCGGFAMPIRENKAQEIYIVMSGEMMAMYAANNISKGILKYANSGGVRLGGLVCNERQTDKELELAEALAGMLGTRLIHFVPRDNIVQHAELRRMTVIEFAPDSKQANEYRQLANKIHNNAGNGTIPTPITMDQLEDLLMEHGIMSAIDESQVGKTASELTA

>Herbaspirillum seropedicae SmR1

MASLRQIAFYGKGGIGKSTTSQNTLAALAQMGQRILIVGCDPKADSTRLILHAKAQDTILSLAADAGSVEDLELEDVMKIGYQNIRCVESGGPEPGVGCAGRGVITSINFLEEEGAYDDTDYVSYDVLGDVVCGGFAMPIRENKAQEIYIVMSGEMMAMYAANNISKGILKYANSGGVRLGGLICNERKTDKELELATALAAKLNSKLIHFVPRDNIVQHAELRRMTVLEYAPDSKQAGEYRTLANKIHENAGNGTIPTPITMDELEDLLMQHGLMPVVDESMVGKSAEVAA

>Azorhizobium caulinodans ORS 571

MSSLRQIAFYGKGGIGKSTTSQNTLAALAEMGHRILIVGCDPKADSTRLILHAKAQDTILSLAAAAGSVEDLELEEVMKIGYRDIRCVESGGPEPGVGCAGRGVITSINFLEENGAYEDIDYVSYDVLGDVVCGGFAMPIRENKAQEIYIVMSGEMMAMYAANNISKGILKYANSGGVRLGGLVCNERQTDKELELAENLAKKLGTQLIYFVPRDNIVQHAELRRMTVIEYAPDSVQANHYRNLAERVHNNGGKGIIPTPITMDELEDMLMEHGIMKTVDESQVGKTAAELAALSA

>Azotobacter vinelandii DJ

MAMRQCAIYGKGGIGKSTTTQNLVAALAEMGKKVMIVGCDPKADSTRLILHSKAQNTIMEMAAEAGTVEDLELEDVLKAGYGGVKCVESGGPEPGVGCAGRGVITAINFLEEEGAYEDDLDFVFYDVLGDVVCGGFAMPIRENKAQEIYIVCSGEMMAMYAANNISKGIVKYANSGSVRLGGLICNSRNTDREDELIIALANKLGTQMIHFVPRDNVVQRAEIRRMTVIEYDPKAKQADEYRALARKVVDNKLLVIPNPITMDELEELLMEFGIMEVEDESIVGKTAEEV

>Azoarcus olearius DQS4

MAKLRQCAIYGKGGIGKSTTTQNLVAALAEAGKKVMIVGCDPKADSTRLILHSKAQTTVMHLAAEAGSVEDLELDDVLSVGFGGVKCVESGGPEPGVGCAGRGVITAINFLEEEGAYDDELDFVFYDVLGDVVCGGFAMPIRENKAQEIYIVCSGEMMAMYAANNIAKGIVKYANSGGVRLGGLICNSRNTDREDELIEALAAAMGTQMIHFVPRDNAVQHAEIRRMTVIEYDPTHKQADQYRQLAQKVLNNKMLVIPTPIEMEQLEALLMEFGIMEQEDESIVGQTAAELAAGAAA

>Ciceribacter sichuanensis S153

MAALRQIAFYGKGGIGKSTTSQNTLAALVDLGQKILIVGCDPKADSTRLILNSKAQDTVLSLAAQEGSVEDLELEDVLKIGYKGIKCVESGGPEPGVGCAGRGVITSINFLEENGAYDDVDYVSYDVLGDVVCGGFAMPIRENKAQEIYIVMSGEMMALYAANNIAKGILKYANSGGVRLGGLICNERQTDRELDLAEALSARLNSKLIHFVPRDNIVQHAELRKQTVIEYAPNSQQAQEYRQLAQKIHDNSGKGTIPTPITMEELEDMLLAFGIMKTDEQMLAELAAKEAAKQAVAAV

>Gluconacetobacter diazotrophicus PA1 A WP_012222808.1

MSKLRQIAFYGKGGIGKSTTSQNTLAALVEMGQKILIVGCDPKADSTRLILNAKAQDTVLSLAAEAGSVEDLELEDVLKIGYKGIKCVESGGPEPGVGCAGRGVITSINFLEENGAYDDVDYVSYDVLGDVVCGGFAMPIRENKAQEIYIVMSGEMMALYAANNIAKGILKYAHSGGVRLGGLICNERQTDREYDLADALAKRLNSKLVHFVPRANIVQHAELRKQTVIEYAPDSAQAGEYRTLAQKIHANSGQGTVPTPITMEELEDMLLEFGIMKTDEQALAELAAKEAKAAAALA

>Martelella alba BGMRC 2036 WP_141150239.1

MSGLRQIAFYGKGGIGKSTTSQNTLAALVDLGQKILIVGCDPKADSTRLILNSKAQDTVLHLAAQEGSVEDLELEDVLKMGYKGIKCVESGGPEPGVGCAGRGVITSINFLEENGAYDDVDYVSYDVLGDVVCGGFAMPIRENKAQEIYIVMSGEMMALYAANNIAKGILKYANSGGVRLGGLICNERQTDRELDLAEALAKRLNSKLIHFVPRDNIVQHAELRKQTVIQYAPNSQQAEEYRQLAQKIHNNSGQGTIPTPITMEELEDMLLDFGIMKTDEQMLAELAAKETAAE

>Mesorhizobium japonicum MAFF 303099 WP_010913608.1

MSGLRQIAFYGKGGIGKSTTSQNTLAALVDLGQKILIVGCDPKADSTRLILNAKAQDTVLHLAAQEGSVEDLELQDVLKIGYKDIKCVESGGPEPGVGCAGRGVITSINFLEENGAYDNVDYVSYDVLGDVVCGGFAMPIRENKAQEIYIVMSGEMMALYAANNIAKGILKYAHSGGVRLGGLICNERQTDRELDLSEALAARLNSKLIHFVPRDNIVQHAELRKMTVIQYAPDSKQAGEYRALAEKIHGNSGQGTIPTPITMEELEDMLLDFGIMKTDEQMLAELQAKEAKLAVAQ

>Rhizobiales AFS011520 WP_112627312.1

MSGLRQIAFYGKGGIGKSTTSQNTLAALVDLGQKILIVGCDPKADSTRLILNSKAQDTVLSLAAQEGSVEDLELEDVLKIGYKGIKCVESGGPEPGVGCAGRGVITSINFLEENGAYDDVDYVSYDVLGDVVCGGFAMPIRENKAQEIYIVMSGEMMALYAANNIAKGILKYANSGGVRLGGLICNERQTDRELDLAEALAARLNSKLMHFVPRDNIVQHAELRKQTVIEYAPNSQQAQEYRQLAQKIHNNSGKGTIPTPITMEELEDMLLDFGIMKTDEQMLAELAAKESAAAH

>Rhizobiales AFS095513 WP_112829892.1

MTALRQIAFYGKGGIGKSTTSQNTLAALVDLGQKILIVGCDPKADSTRLILNSKAQDTVLSLAAQEGSVEDLELEDVLKIGYKGIKCVESGGPEPGVGCAGRGVITSINFLEENGAYDDVDYVSYDVLGDVVCGGFAMPIRENKAQEIYIVMSGEMMALYAANNIAKGILKYANSGGVRLGGLICNERQTDRELDLAEALAARLNSKLIHFVPRDNIVQHAELRKQTVIEYAPNSQQAQEYRQLAQKIHDNSGKGTIPTPITMEELEDMLLAFGIMKTDEQMLAELAAKEAAAAAH

>Rhizobium populi CCTCC AB 2013068 Ga0196655_102249

MAALRQIAFYGKGGIGKSTTSQNTLAALVDLGQKILIVGCDPKADSTRLILNSKAQDTVLSLAAQEGSVEDLELEDVLKVGYKGIKCVESGGPEPGVGCAGRGVITSINFLEENGAYDDVDYVSYDVLGDVVCGGFAMPIRENKAQEIYIVMSGEMMALYAANNIAKGILKYANSGGVRLGGLICNERQTDRELDLAEALSARLNSKLIHFVPRDNIVQHAELRKQTVIEYAPNSQQAQEYRQLARKIHDNSGKGTIPTPITMEELEDMLLAFGIMKTDEQMLAELAAKEAVAPAAAV
