# Supplementary material for: Non-nodulating Paenirhizobium fix nitrogen in association with cereal roots
Source: ISME Commun. 2026 Jun 24;6(1):ycag156. doi: 10.1093/ismeco/ycag156 (PMC13455631; doi:10.1093/ismeco/ycag156)
Supplement: Supplementary file 1 [file supplementary-material_ycag156.zip › Supplementary methods.docx]

**list2matrix.py**

--------------------------------------------------------------------------------------------------------------------------------------

import pandas as pd

import numpy as np

\# Load your data into a DataFrame

data = pd.read_csv("ezaai_ciceribacter.tsv", delim_whitespace=True, header=0)

\# Assume columns 3 and 4 are 0-indexed (e.g., Python indexing)

strain1 = data.iloc[:, 2] # Column 3 (Strain A)

strain2 = data.iloc[:, 3] # Column 4 (Strain B)

similarity = data.iloc[:, 4] # Similarity values (Column 5)

\# Identify unique strains from both strain1 and strain2 columns

unique_strains = np.unique(np.concatenate([strain1, strain2]))

\# Create a dictionary to map strain names to matrix indices

strain_index = {strain: idx for idx, strain in enumerate(unique_strains)}

\# Initialize an empty similarity matrix

similarity_matrix = np.zeros((len(unique_strains), len(unique_strains)))

\# Loop over each pair of strains and fill the similarity matrix

for i in range(len(data)):

s1 = strain1.iloc[i]

s2 = strain2.iloc[i]

sim = similarity.iloc[i]

\# Get indices of the strains

row_idx = strain_index[s1]

col_idx = strain_index[s2]

\# Fill the matrix symmetrically (for undirected pairings)

similarity_matrix[row_idx, col_idx] = sim

similarity_matrix[col_idx, row_idx] = sim

\# Create a DataFrame for better visualization

similarity_df = pd.DataFrame(similarity_matrix, index=unique_strains, columns=unique_strains)

\# Save the matrix to a CSV or display it

similarity_df.to_csv("similarity_matrix.csv")

print(similarity_df)

--------------------------------------------------------------------------------------------------------------------------------------

**sim2distlow.py**

--------------------------------------------------------------------------------------------------------------------------------------

import pandas as pd

import numpy as np

\# Load the similarity matrix

similarity_df = pd.read_csv("similarity_matrix.csv", index_col=0)

\# Convert similarity to distance: distance = 1 - (similarity / 100)

distance_matrix = 1 - (similarity_df / 100)

\# Set diagonal values to NaN (optional, if you want to leave them empty)

np.fill_diagonal(distance_matrix.values, np.nan)

\# Create a mask for the upper triangular part, including the diagonal

mask = np.triu(np.ones(distance_matrix.shape), k=1)

\# Apply the mask to set the upper triangular part to NaN

distance_matrix[mask == 1] = np.nan

\# Format the matrix to 4 decimal places

distance_matrix = distance_matrix.round(4)

\# Save the output to a new file

distance_matrix_df = pd.DataFrame(distance_matrix, index=similarity_df.index, columns=similarity_df.columns)

distance_matrix_df.to_csv("lower_triangular_distance_matrix_4decimals.csv")

\# Print the result (optional)

print(distance_matrix_df)

--------------------------------------------------------------------------------------------------------------------------------------
