# Supplementary material for: Non-nodulating Paenirhizobium fix nitrogen in association with cereal roots
Source: ISME Commun. 2026 Jun 24;6(1):ycag156. doi: 10.1093/ismeco/ycag156 (PMC13455631; doi:10.1093/ismeco/ycag156)
Supplement: Supplementary file 1 [file supplementary-material_ycag156.zip › Supplementary Table 1. list of strains and plasmids.docx]

**Supplementary table 1 |** List of bacterial strains and plasmids used in this study.

| **Strain** | **Description** | **Source** |
| --- | --- | --- |
| *Paenirhizobium* sp. B6 | Alphaproteobacterium isolated from barley roots | This study |
| *Paenirhizobium* sp. J2 | Alphaproteobacterium isolated from barley roots | This study |
| B6-GFP | *C. sichuanensis* B6 tagged with pTn*7*-SCOUT11.G plasmid | This study |
| J2-GFP | *C*.*sichuanensis* J2 tagged with pTn*7*-SCOUT11.G plasmid | This study |
| DH5α | Competent *E. coli* strain for use in transformations, carrying the following mutations: F^-^ *deoR* *endA1* *recA1 relA1 gyrA96 hsdR17*(rk^-^mk^+^) *supE44 thi-1-phoA* Δ(*lacZYA-argF*) *ΔlacU169* φ80*lacZDM15* l. | Bioline |
| **Plasmids** | **Description** | **Source** |
| pTNS3 | Transposase delivery plasmid for integration of mini-Tn*7* cassettes | (Choi & Schweizer, 2006) |
| pRK2013 | Helper plasmid for triparental matings with Km^20^/Neo^80^ resistance | (Ditta et al., 1980) |
| pTn*7*-SCOUT11.G | Low copy number suicide plasmid with Andersons J23104-RBstd-sfGFP-DT16-pOGG276 (pUC18R6K-miniTn*7*T-Gm) | (Jorrin et al., 2024) |
