# Supplementary material for: Non-nodulating Paenirhizobium fix nitrogen in association with cereal roots
Source: ISME Commun. 2026 Jun 24;6(1):ycag156. doi: 10.1093/ismeco/ycag156 (PMC13455631; doi:10.1093/ismeco/ycag156)
Supplement: Supplementary file 1 [file supplementary-material_ycag156.zip › Supplementary Table 8 ANI.docx]

**Supplementary table 8.** ANI values comparing nucleotide sequence similarity against other Rhizobiaceae members.

|  | Strain | B6 | J2 |  |  |
| --- | --- | --- | --- | --- | --- |
|  | B6 | 100.00 | 98.30 |  |  |
|  | J2 | 98.36 | 100.00 |  |  |
|  | *Ciceribacter sichuanensis* S101^TS^ | 98.18 | 98.14 |  |  |
|  | *Paenirhizobium daejeonense* L61^T*^ | 92.70 | 92.84 |  |  |
|  | *Rhizobium cremeum* W15^T^ | 88.23 | 88.20 |  |  |
|  | *Rhizobium terricola* S-51^T^ | 82.03 | 81.88 |  |  |
|  | *Paenirhizobium selenitireducens* ATCC BAA-1503^T^ | 81.97 | 81.92 |  |  |
|  | *Ciceribacter ferrooxidans* F8825^T^ | 80.53 | 80.54 |  |  |
|  | *Ciceribacter lividus* DSM 25528^T*^ | 80.51 | 80.53 |  |  |
|  | *Paenirhizobium naphthalenivorans* NBRC 107585^T^ | 80.47 | 80.45 |  |  |
|  | *Ciceribacter thiooxidans* F43B^T^ | 80.19 | 80.26 |  |  |
|  | *Peteryoungia ipomeae* shin9-1^T*^ | 79.96 | 79.91 |  |  |
|  | *Pseudorhizobium pelagicum* R1-200B4^T*^ | 79.52 | 79.41 |  |  |
|  | *Neorhizobium galegae* HAMBI540 ^T*^ | 79.41 | 79.48 |  |  |
|  | *Rhizobium leguminosarum* USDA2370 ^T*^ | 79.19 | 79.18 |  |  |
|  | *Fererhizobium litorale* KMM 9576 ^T*^ | 79.15 | 79.14 |  |  |
|  | *Allorhizobium undicola* LMG 11875 ^T*^ | 78.77 | 78.75 |  |  |
|  | *Agrobacterium tumefaciens* ATCC 4720 ^T*^ | 78.77 | 78.83 |  |  |
|  | T Type strain. T* Type strain that defines the genus |  |  |  |  |
