# Supplementary material for: Non-nodulating Paenirhizobium fix nitrogen in association with cereal roots
Source: ISME Commun. 2026 Jun 24;6(1):ycag156. doi: 10.1093/ismeco/ycag156 (PMC13455631; doi:10.1093/ismeco/ycag156)
Supplement: Supplementary file 1 [file supplementary-material_ycag156.zip › Supplementary Table 9.docx]

**Supplementary Table 9 |** List of different carbon sources found on Biolog PM1 MicroPlates^TM^. The list contains details on the carbon composition and type for each compound and whether Ciceribacter spp. B6 or J2 were able to catabolise the carbon source (+, growth; – , no growth).

| Carbon source | Number of carbon atoms | Type of carbon source | B6 growth | J2 growth |
| --- | --- | --- | --- | --- |
| Glucuronamide | Variable carbons | Amide | - | - |
| Phenylethyl-amine | 8 carbons | Amine | - | - |
| 2-Aminoethanol | 2 carbons | Amine | + | - |
| Tyramine | 8 carbons | Amine | - | - |
| L-Aspartic Acid | 4 carbons | Amino acid | - | + |
| L-Proline | 5 carbons | Amino acid | + | + |
| D-Alanine | 3 carbons | Amino acid | - | + |
| D-Serine | 3 carbons | Amino acid | - | + |
| L-Glutamic Acid | 5 carbons | Amino acid | + | + |
| L-Asparagine | Variable carbons | Amino acid | + | + |
| D-Aspartic Acid | 4 carbons | Amino acid | - | - |
| L-Glutamine | 5 carbons | Amino acid | + | + |
| D-Threonine | 4 carbons | Amino acid | - | - |
| L-Serine | 3 carbons | Amino acid | - | + |
| L-Threonine | 4 carbons | Amino acid | - | - |
| L-Alanine | 3 carbons | Amino acid | + | - |
| N-Acetyl-D-Glucosamine | 8 carbons | Amino sugar | - | + |
| N-Acetyl-β-D-Mannosamine | Variable carbons | Amino sugar | - | + |
| Succinic Acid | 4 carbons | Dicarboxylic acid | + | + |
| Glycyl-L-Aspartic Acid | Variable carbons | Dipeptide | - | + |
| Glycyl-L-Glutamic Acid | Variable carbons | Dipeptide | - | + |
| L-Alanyl-Glycine | Variable carbons | Dipeptide | + | + |
| Glycyl-L-Proline | Variable carbons | Dipeptide | + | + |
| D-Trehalose | 12 carbons | Disaccharide sugar | + | + |
| Maltose | 12 carbons | Disaccharide sugar | + | + |
| D-Melibiose | 12 carbons | Disaccharide sugar | - | + |
| Lactulose | 12 carbons | Disaccharide sugar | - | - |
| Sucrose | 12 carbons | Disaccharide sugar | + | - |
| ɑ-D-Lactose | 12 carbons | Disaccharide sugar | + | + |
| D-Cellobiose | 12 carbons | Disaccharide sugar | + | + |
| D-Mannose | 6 carbons | Monosaccharide sugar | + | + |
| D-Galactose | 6 carbons | Monosaccharide sugar | + | + |
| L-Fucose | 6 carbons | Monosaccharide sugar | + | + |
| L-Rhamnose | 6 carbons | Monosaccharide sugar | + | + |
| D-Fructose | 6 carbons | Monosaccharide sugar | + | + |
| ɑ-D-Glucose | 6 carbons | Monosaccharide sugar | + | + |
| ɑ-Methyl-D-Galactoside | 6 carbons | Monosaccharide sugar | - | + |
| β-Methyl-D-Glucoside | 6 carbons | Monosaccharide sugar | - | + |
| D-Psicose | 6 carbons | Monosaccharide sugar | - | - |
| Thymidine | 10 carbons | Nucleoside | - | - |
| Uridine | Variable carbons | Nucleoside | + | + |
| 2-Deoxy Adenosine | Variable carbons | Nucleoside | - | + |
| Adenosine | Variable carbons | Nucleoside | - | + |
| Inosine | Variable carbons | Nucleoside | - | + |
| D-Saccharic Acid | 6 carbons | Organic acid | - | + |
| Formic Acid | 1 carbon | Organic acid | + | + |
| D-Glucuronic Acid | 6 carbons | Organic acid | - | + |
| D-Gluconic Acid | 6 carbons | Organic acid | - | + |
| L-Lactic Acid | 3 carbons | Organic acid | + | + |
| D-Galactonic Acid-Ɣ-Lactone | 6 carbons | Organic acid | - | + |
| D-L-Malic Acid | 4 carbons | Organic acid | - | + |
| Acetic Acid | 2 carbons | Organic acid | + | + |
| D-Glucosaminic Acid | 6 carbons | Organic acid | - | - |
| ɑ-Keto-Glutaric Acid | 5 carbons | Organic acid | - | - |
| ɑ-Keto-Butyric Acid | 4 carbons | Organic acid | - | - |
| m-Tartaric Acid | 4 carbons | Organic acid | - | - |
| ɑ-Hydroxy Glutaric Acid-ɣ-Lactone | 5 carbons | Organic acid | - | - |
| ɑ-Hydroxy Butyric Acid | 4 carbons | Organic acid | - | - |
| Glyoxylic Acid | 2 carbons | Organic acid | - | - |
| Citric Acid | 6 carbons | Organic acid | - | + |
| Fumaric Acid | 4 carbons | Organic acid | + | + |
| Bromo Succinic Acid | 4 carbons | Organic acid | + | + |
| Propionic Acid | 3 carbons | Organic acid | + | + |
| Glycolic Acid | 2 carbons | Organic acid | - | - |
| D-Malic Acid | 4 carbons | Organic acid | + | + |
| L-Malic Acid | 4 carbons | Organic acid | + | + |
| Acetoacetic Acid | 4 carbons | Organic acid | - | - |
| Mono Methyl Succinate | 5 carbons | Organic acid | + | + |
| D-Galacturonic Acid | 6 carbons | Organic acid | - | + |
| p-Hydroxy Phenyl Acetic Acid | 8 carbons | Organic acid | - | + |
| m-Hydroxy Phenyl Acetic Acid | 8 carbons | Organic acid | - | + |
| Pyruvic Acid | 3 carbons | Organic acid | + | + |
| L-Galactonic Acid-ɣ-Lactone | 6 carbons | Organic acid | - | + |
| D-L-ɑ-Glycerol-Phosphate | 3 carbons | Organic compound | - | + |
| D-Glucose-6-Phosphate | 6 carbons | Organic compound | - | + |
| 1,2-Propanediol | 3 carbons | Organic compound | - | - |
| D-Glucose-1-Phosphate | 6 carbons | Organic compound | - | - |
| D-Fructose-6-Phosphate | 6 carbons | Organic compound | - | + |
| Methyl Pyruvate | 3 carbons | Organic compound | + | + |
| L-Arabinose | 5 carbons | Pentose sugar | + | + |
| D-Xylose | 5 carbons | Pentose sugar | + | + |
| D-Ribose | 5 carbons | Pentose sugar | + | + |
| L-Lyxose | 5 carbons | Pentose sugar | - | + |
| Tween 20 | Variable carbons | Polysorbate surfactant | - | - |
| Tween 40 | Variable carbons | Polysorbate surfactant | - | - |
| Tween 80 | Variable carbons | Polysorbate surfactant | - | - |
| Mucic Acid | 6 carbons | Sugar acid | - | + |
| Dulcitol | 6 carbons | Sugar alcohol | + | - |
| D-Mannitol | 6 carbons | Sugar alcohol | + | - |
| D-Sorbitol | 6 carbons | Sugar alcohol | + | + |
| Adonitol | 5 carbons | Sugar alcohol | - | - |
| m-Inositol | 6 carbons | Sugar alcohol | + | + |
| Tricarballylic Acid | 6 carbons | Tricarboxylic acid | - | - |
| Glycerol | 3 carbons | Triol alcohol | + | + |
| Maltotriose | 18 carbons | Trisaccharide sugar | - | + |
