# Supplementary material for: Non-nodulating Paenirhizobium fix nitrogen in association with cereal roots
Source: ISME Commun. 2026 Jun 24;6(1):ycag156. doi: 10.1093/ismeco/ycag156 (PMC13455631; doi:10.1093/ismeco/ycag156)
Supplement: Supplementary file 1 [file supplementary-material_ycag156.zip › Supplementary_Figure_2_ycag156.docx]

**Supplementary Figure 4 |** Growth characterisation of C. sichuanensis grown on different media. (**A**)Growth of B6 and J2 seen on UMS with methanol and either with or without LaCl_3_ after 7 days of growth. (**B**) B6 and J2 growth on UMS^succ^ plates supplemented with all three B-vitamins and then each in isolation after 3 days of incubation at 28˚C.

(**A**)

(**B**)
