# Supplementary material for: Non-nodulating Paenirhizobium fix nitrogen in association with cereal roots
Source: ISME Commun. 2026 Jun 24;6(1):ycag156. doi: 10.1093/ismeco/ycag156 (PMC13455631; doi:10.1093/ismeco/ycag156)
Supplement: Supplementary file 1 [file supplementary-material_ycag156.zip › Supplementary_Table_2_ycag156.docx]

**Supplementary Table – primers used**

| Primer name | Direction | Sequence (5' to 3') | Target gene | DNA Amplicon size (bp) | Tm (°C) | Reference |
| --- | --- | --- | --- | --- | --- | --- |
| 27F1 | F | ACACACNNNAGAGTTTGATCCTGGCTCAG | 16s rRNA | 1500 | 55 | - |
| 27F2 | F | TGTGTGNNNAGAGTTTGATCCTGGCTCAG | 16s rRNA | 1500 | 55 | - |
| 27F3 | F | GAGAGANNNAGAGTTTGATCCTGGCTCAG | 16s rRNA | 1500 | 55 | - |
| 27F4 | F | CTCTCTNNNAGAGTTTGATCCTGGCTCAG | 16s rRNA | 1500 | 55 | - |
| 27F5 | F | ATCGATNNNAGAGTTTGATCCTGGCTCAG | 16s rRNA | 1500 | 55 | - |
| 27F6 | F | TCAACANNNAGAGTTTGATCCTGGCTCAG | 16s rRNA | 1500 | 55 | - |
| 27F7 | F | GCTACGNNNAGAGTTTGATCCTGGCTCAG | 16s rRNA | 1500 | 55 | - |
| 27F8 | F | CGTTAGNNNAGAGTTTGATCCTGGCTCAG | 16s rRNA | 1500 | 55 | - |
| 27F9 | F | AGCAGCNNNAGAGTTTGATCCTGGCTCAG | 16s rRNA | 1500 | 55 | - |
| 27F10 | F | TAAGGTNNNAGAGTTTGATCCTGGCTCAG | 16s rRNA | 1500 | 55 | - |
| 1492R1 | R | GTGTGTNNNACGGYTACCTTGTTACGACTT | 16s rRNA | 1500 | 55 | - |
| 1492R2 | R | CACACANNNACGGYTACCTTGTTACGACTT | 16s rRNA | 1500 | 55 | - |
| 1492R3 | R | TCTCTCNNNACGGYTACCTTGTTACGACTT | 16s rRNA | 1500 | 55 | - |
| 1492R4 | R | AGAGAGNNNACGGYTACCTTGTTACGACTT | 16s rRNA | 1500 | 55 | - |
| 1492R5 | R | ATCGATNNNACGGYTACCTTGTTACGACTT | 16s rRNA | 1500 | 55 | - |
| 1492R6 | R | TGTTGANNNACGGYTACCTTGTTACGACTT | 16s rRNA | 1500 | 55 | - |
| 1492R7 | R | CGTAGCNNNACGGYTACCTTGTTACGACTT | 16s rRNA | 1500 | 55 | - |
| 1492R8 | R | CTAACGNNNACGGYTACCTTGTTACGACTT | 16s rRNA | 1500 | 55 | - |
| 1492R9 | R | GCTGCTNNNACGGYTACCTTGTTACGACTT | 16s rRNA | 1500 | 55 | - |
| 1492R10 | R | ACCTTANNNACGGYTACCTTGTTACGACTT | 16s rRNA | 1500 | 55 | - |
| DVV | R | ATI GCR AAI CCI CCR CAI ACI ACR TC | nifH | 394 | 55 | Ando et al., 2005  Angel et al, 2018 |
| IKG3 | F | GCI WTH TAY GGI AAR GGI GGI ATH GGI AA 19–47 | nifH | 394 | 55 | Ando et al., 2005  Angel et al, 2018 |
